# A Synergistic Genetic Engineering Strategy Induced Triacylglycerol Accumulation in Potato (*Solanum tuberosum*) Leaf

**DOI:** 10.3389/fpls.2020.00215

**Published:** 2020-03-06

**Authors:** Xiao-yu Xu, Sehrish Akbar, Pushkar Shrestha, Lauren Venugoban, Rosangela Devilla, Dawar Hussain, Jiwon Lee, Melanie Rug, Lijun Tian, Thomas Vanhercke, Surinder P. Singh, Zhongyi Li, Peter J. Sharp, Qing Liu

**Affiliations:** ^1^CSIRO Agriculture and Food, Canberra, ACT, Australia; ^2^Plant Breeding Institute and Sydney Institute of Agriculture, School of Life and Environmental Sciences, The University of Sydney, Camperdown, NSW, Australia; ^3^Center for Advanced Microscopy, The Australian National University, Canberra, ACT, Australia

**Keywords:** potato, *Solanum tuberosum*, lipids, triacylglycerol, lipid droplets, plastoglobuli

## Abstract

Potato is the 4th largest staple food in the world currently. As a high biomass crop, potato harbors excellent potential to produce energy-rich compounds such as triacylglycerol as a valuable co-product. We have previously reported that transgenic potato tubers overexpressing *WRINKLED1*, *DIACYLGLYCEROL ACYLTRANSFERASE 1*, and *OLEOSIN* genes produced considerable levels of triacylglycerol. In this study, the same genetic engineering strategy was employed on potato leaves. The overexpression of *Arabidopsis thaliana WRINKED1* under the transcriptional control of a senescence-inducible promoter together with *Arabidopsis thaliana DIACYLGLYCEROL ACYLTRANSFERASE 1* and *Sesamum indicum OLEOSIN* driven by the *Cauliflower Mosaic Virus 35S* promoter and small subunit of Rubisco promoter respectively, resulted in an approximately 30- fold enhancement of triacylglycerols in the senescent transgenic potato leaves compared to the wild type. The increase of triacylglycerol in the transgenic potato leaves was accompanied by perturbations of carbohydrate accumulation, apparent in a reduction in starch content and increased total soluble sugars, as well as changes of polar membrane lipids at different developmental stages. Microscopic and biochemical analysis further indicated that triacylglycerols and lipid droplets could not be produced in chloroplasts, despite the increase and enlargement of plastoglobuli at the senescent stage. Possibly enhanced accumulation of fatty acid phytyl esters in the plastoglobuli were reflected in transgenic potato leaves relative to wild type. It is likely that the plastoglobuli may have hijacked some of the carbon as the result of *WRINKED1* expression, which could be a potential factor restricting the effective accumulation of triacylglycerols in potato leaves. Increased lipid production was also observed in potato tubers, which may have affected the tuberization to a certain extent. The expression of transgenes in potato leaf not only altered the carbon partitioning in the photosynthetic source tissue, but also the underground sink organs which highly relies on the leaves in development and energy deposition.

## Introduction

Plant vegetative tissues such as leaves are usually viewed as ‘source organs,’ within which a matrix of assimilative photosynthetic activities and metabolite transport proceeds. Other tissues like seeds, fruits and tubers are considered as ‘sink organs’ because of their predominant functions in nutrient and energy storage (Fischer and Weber, 2002). The storage substances reserved in the sink organs are important to seed germination or sprouting and subsequent seedling establishment, whilst serving as the major economic products in agricultural production (Poxleitner et al., 2006; Graham, 2008; Yang et al., 2009; Kelly et al., 2011). Conventionally, following harvesting, the leftover vegetative biomass is either used as livestock fodder or bio-fertilizer, if not wasted. Recently the possibility to take further advantage of high biomass plants as a source of biodiesel was suggested (Vanhercke et al., 2013, 2019), as manipulation of plant metabolic networks through genetic engineering approaches has provided the insight that some non-sink plant vegetative tissues may be reprogrammed to store energy-dense compounds such as oil (Chapman et al., 2013; Xu and Shanklin, 2016).

Triacylglycerols (TAG), as the major form of oil in plants, store higher levels of energy compared to starch and cellulose and have long been regarded as the most applicable alternative feedstock of fossil fuels (Durrett et al., 2008; Carlsson et al., 2011; Chapman and Ohlrogge, 2012). Oil palm (*Elaeis guineensis*) and oilseed crops including soybean (*Glycine max*), rapeseed (*Brassica napus*) and sunflower (*Helianthus annuus*) are the current main production platforms of vegetable oils (Xu et al., 2018; Vanhercke et al., 2019). However, the ratio of oil-bearing seeds to the whole plant biomass is often small in such crops, suggesting a possibly viable value in engineering the vegetative biomass plant parts for TAG production. Presently, the functional annotations of many genes involved in lipid metabolism have been made available in model plant *Arabidopsis thaliana* and several oil-rich plant species (Costa et al., 2010; Bourgis et al., 2011; Brown et al., 2012; Nguyen et al., 2013; Higashi et al., 2015). With the increased knowledge of TAG biosynthesis and turnover, it has become possible to genetically modify oil production and accumulation in plant vegetative tissues. Whilst the current understanding of plant TAG metabolism is mostly derived from studies on oilseeds, the biochemical pathways and their regulatory mechanisms are relatively conserved between seed and vegetative plant tissues (Xu and Shanklin, 2016).

As a result, a suite of key genes regulating TAG metabolism has been identified and tested in model plants as well as some potential platform plants, showing that a multigene-based pathway manipulation of oil production in plant vegetative tissues could be feasible. Up to 15% TAG in leaf dry weight (DW) was accumulated in transgenic tobacco (*Nicotiana tabacum*) through the simultaneous overexpression of *A. thaliana WRINKLED1* (*AtWRI1*), *A. thaliana diacylglycerol acyltransferase1* (*AtDGAT1*) and sesame (*Sesamum indicum*) *OLEOSIN1* (*SiOLEOSIN1*) genes (Vanhercke et al., 2014a), coined the ‘Push, Pull and Protect’ synergistic strategy for oil increase (Vanhercke et al., 2014b). In addition, the C_4_ plant sorghum (*Sorghum bicolor*) was recently reported to produce between 3 and 8.4% of TAG by DW in vegetative tissues following co-expression of *Zea mays WRI1, Umbelopsis ramanniana DGAT2a*, and *SiOLEOSIN* (Vanhercke et al., 2018). Further enhancement of TAG accumulation was achieved by downregulating the TAG-specific lipase sugar-dependent 1 (*SDP1*) gene, which resulted in doubled TAG production (30% of DW) in transgenic tobacco leaf (Vanhercke et al., 2017), while sugarcane (*Saccharum officinarum*) engineered with the similar methodology also displayed a 95- fold enhancement of TAG content in vegetative tissues (Zale et al., 2016).

Potato is traditionally regarded as a vegetable food rich in starch. With a global production of 388 million tonnes in 2017, potato is currently the 4^*th*^ largest staple food in the world (Zaheer and Akhtar, 2016). There is no doubt that the exploration of new opportunities to add value to the potato crop would be of potential benefit. We have previously applied the ‘Push, Pull and Protect’ strategy in potato through tuber-specific expression of *AtWRI1* driven by the patatin promoter, together with *AtDGAT1* and *SiOLEOSIN1* which led to an almost 100-fold increase in TAG levels in tuber tissues (Liu et al., 2017a). TAG increase to a lesser extent in potato tuber has also been observed when *AtWRI1* was overexpressed alone under transcriptional control of an alternative tuber-specific promoter derived from the granule bound starch synthase (*GBSS*) gene (Hofvander et al., 2016). A rather moderate increase in TAG in potato tuber was displayed through the overexpression of *A. thaliana* acetyl-CoA carboxylase (*ACCase*) (Klaus et al., 2004). As a source organ, potato leaf represents the greatest proportion of the aerial vegetative tissues, and primarily provides apoplastic sucrose to support the underground tuber growth (Fernie and Willmitzer, 2001; Hastilestari et al., 2018). Earlier research results on potato leaf molecular biology/biochemistry were mostly focused on pest control (Douches et al., 2001; Dita Rodriguez et al., 2006; Peiman and Xie, 2006; Athanikar and Badar, 2016) and photosynthetic regulation (Fleisher et al., 2006; Timlin et al., 2006; Rolando et al., 2015; Paradiso et al., 2018). The potential of potato leaves as a biofactory for TAG production is highly attractive considering the size of the aboveground biomass as a byproduct of potato production. However, earlier attempts failed to enhance TAG accumulation in potato leaf by transforming the ‘Push, Pull, Protect’ construct which was successfully used in generating high oil tobacco leaf (Vanhercke et al., 2014a), likely due to the strong pleiotropic effects of *AtWRI1* expression driven by the green tissue active promoter derived from the small subunit of Rubisco (*SSU*) gene (Qing Liu, unpublished data).

In this study, a senescence-inducible promoter, the Senescence Associated Gene 12 (*SAG12*) derived from *A. thaliana* (Noh and Amasino, 1999), was utilized to regulate the expression of *AtWRI1* in combination with *AtDGAT1* controlled by *CaMV-35S* promoter and *SiOLEOSIN1* controlled by *SSU* promoter. The employment of a senescence-inducible promoter in driving *AtWRI1* is anticipated to minimize the potentially undesirable pleiotropic effects of overexpressing *WRI1* on the selection of transgenic cells and subsequent growth and development of transgenic plants (Yang et al., 2015; Kong and Ma, 2018). We were able to increase TAG in potato leaf, and the effects of transgenes on other carbohydrates, mainly total starch/sugars are evaluated. The potential factors limiting the effective accumulation of TAG in transgenic potato leaves were fundamentally explored. Further, the impacts of transgene expression on tuber constituents, morphology and production have also been assessed.

## Results

### Validation and Assessment of Transgene Expressions in Potato Leaf and the Selection of Representative Lines for Further Analysis

**FIGURE 1:**
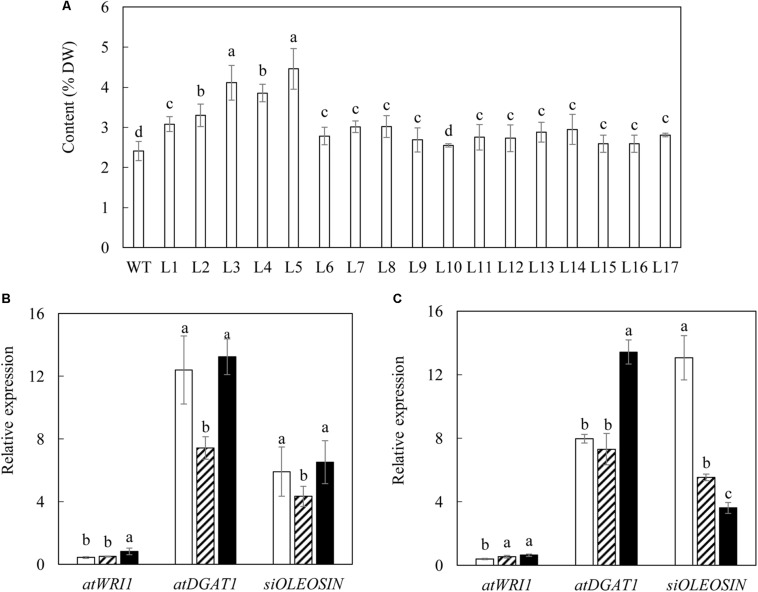
Selection of transgenic potato plants. **(A)** TFA content in the senescent leaves of WT and seventeen selected transgenic potato lines at T_0_ generation. **(B,C)** Real-time qRT-PCR analysis of gene expressions in the leaves of two selected transgenic lines, L3 **(B)** and L5 **(C)**, at the three developmental stages: flowering stage (open bar), mature stage (bar with upward diagonal) and senescent stage (black bar). The data represent the mean values ± standard deviation (SD) of three biological replicates. Letters (a, b, c, and d) above the bars are based on LSD, bars marked with different letters are statistically significantly different at *P* < 0.05.

A total of 17 independent primary transgenic lines were selected *via* the transformation of pOIL076 construct into potato (*Solanum tuberosum* cv Atlantic) on the kanamycin-containing media. The transgenic status of these plants was verified by polymerase chain reaction (PCR) of each of the three transgenes being overexpressed, including *AtWRI1*, *AtDGAT1* and *SiOLEOSIN1* from genomic DNA (data not shown). The analysis of the total fatty acid (TFA) contents of senescent leaves of the 17 independent T_0_ transgenic potatoes showed a significant variation between 2.6 and 4.5% of leaf DW, compared to 2.4% in the wild type (WT) (Figure 1A). Among these transgenic lines, L3 and L5, which contained relatively high levels of TFA, 4.11 and 4.46% respectively, were selected for further analysis. Potato plants are typically propagated vegetatively by tuber-cutting. Consequently the transgenes in transgenic potato plants remain in their heterozygous status without segregation. In order to obtain synchronized growth and physiological status of potato plants for analysis, WT, L3 and L5 transgenic lines were propagated by tuber-cutting and grown under the controlled glasshouse conditions and sampled at three developmental stages including flowering, mature and senescent stages.

Transgene expression assessment was carried out through real-time quantitative reverse transcription polymerase chain reaction (qRT-PCR). The expressions of *AtWRI1*, *AtDGAT1* and *SiOLEOSIN* were not detected in WT, but showed variable expression patterns in the two selected transgenic lines during plant development, relative to the reference gene *S. tuberosum* cyclophilin (*stCYP*) (Figures 1B,C). *AtWRI1* displayed low yet consistent expression in both transgenic lines at the flowering stage, and was significantly increased afterward, particularly at the senescent stage in L3 (Figure 1B). *AtDGAT1* constantly exhibited the highest expression among the three transgenes, which peaked at the senescent stage in both L3 and L5. The expression of *SiOLEOSIN* was relatively consistent over the three developmental stages in L3 (Figure 1B), in contrast to L5 in which the highest expression was observed at the flowering stage (Figure 1C).

### Characterization of Lipids and Carbohydrate Accumulations in Transgenic Lines During Plant Development

**FIGURE 2:**
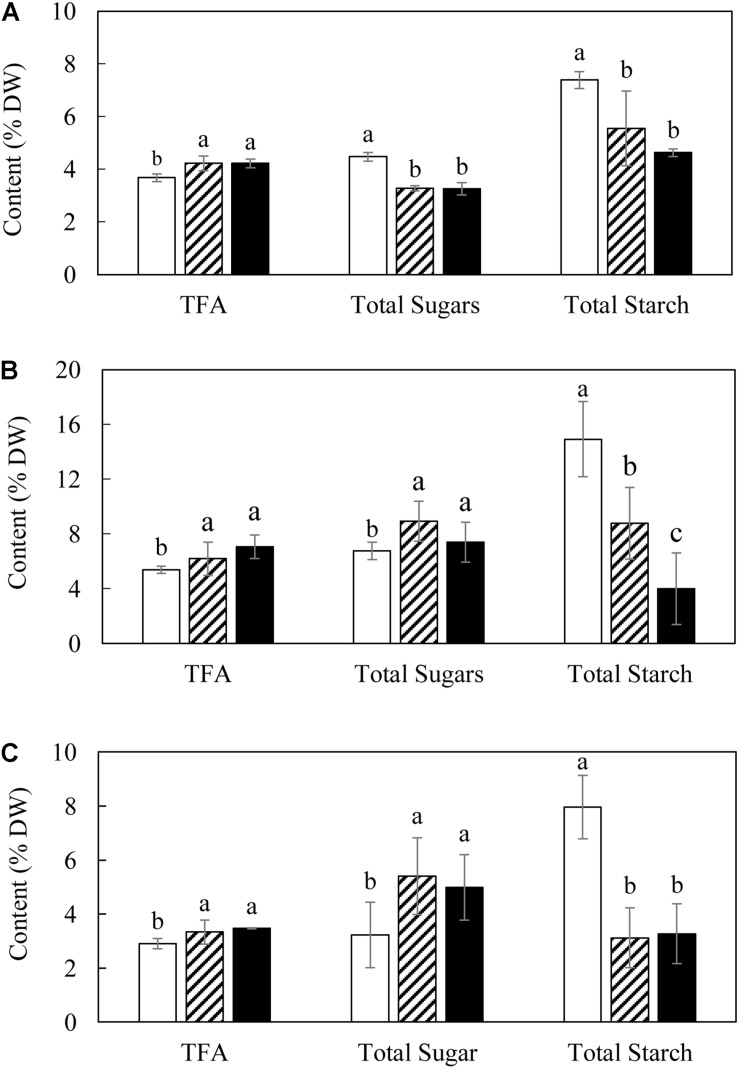
Contents of TFA and total carbohydrate in potato leaves at the three developmental stages in WT (open bar), L3 (bar with upward diagonal) and L5 (black bar). **(A)** Flowering stage; **(B)** Mature stage; **(C)** Senescent stage. The data represent the mean values ± SD of three biological replicates. Letters (a, b, c, and d) above the bars are based on LSD, bars marked with different letters are statistically significantly different at *P* < 0.05.

Total fatty acid contents of both L3 and L5 showed consistently significant increases compared with WT over the three developmental stages, and reached the maximum level at the mature stage as 6.19 and 7.05% of leaf DW respectively (Figure 2B), but reduced thereafter. There were significant variations in the contents of starch and the total soluble sugars between transgenic lines and WT. Specifically, the contents of the total soluble sugars in L3 and L5 showed about 1.4-fold reduction at the flowering stage (Figure 2A), but were significantly increased at the mature and senescent stages relative to WT (Figure 2C). The starch contents of L3 and L5 were consistently lower than WT over the entire growth period. For example, the starch content in L3 was reduced drastically to as low as 3.12% (DW) at the senescent stage, which is a 2.6-fold drop compared to 7.96% in WT (Figure 2C). At the mature stage, starch content in WT reached as high as 14.91% of leaf DW, in contrast to transgenic lines with rather low starch accumulations (8.77% in L3 and 4% in L5, respectively) (Figure 2B).

**FIGURE 3:**
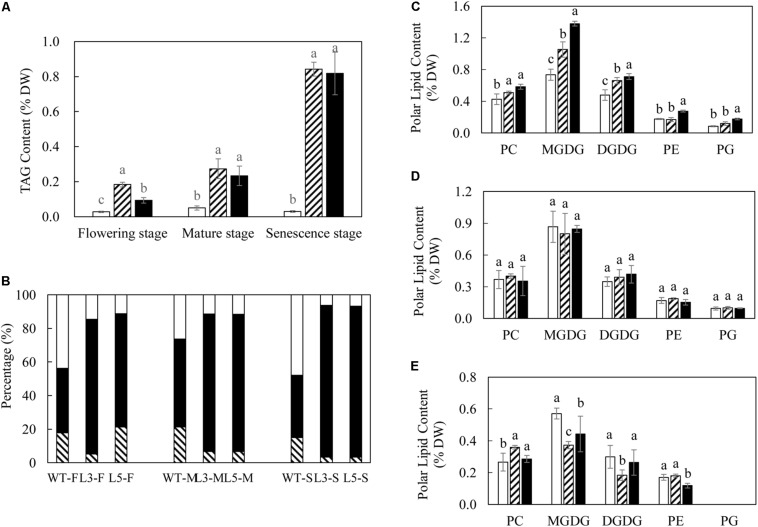
Lipid dynamics in potato leaves at the three developmental stages. **(A)** TAG content in WT (open bar), L3 (bar with upward diagonal) and L5 (black bar); **(B)** Proportional variations of DAG (bar with downward diagonal), TAG (black bar) and FFA (open bar) at the flowering (F), mature (M), and senescent (S) stages. **(C)** Polar lipids content at the flowering stage in WT (open bar), L3 (bar with upward diagonal) and L5 (black bar); **(D)** Polar lipids content at the mature stage in WT (open bar), L3 (bar with upward diagonal) and L5 (black bar); **(E)** Polar lipids content at the senescent stage in WT (open bar), L3 (bar with upward diagonal) and L5 (black bar). The data represent the mean values ± SD of three biological replicates. Letters (a, b, c, and d) above the bars are based on LSD, bars marked with different letters are statistically significantly different at *P* < 0.05.

In comparison to the rather moderate increases in TFA, TAG accumulation in both L3 and L5 was much more evident over the three developmental stages (Figure 3A). The highest TAG contents in L3 and L5 were recorded at the senescent stage as 0.84 and 0.82% of leaf DW respectively, which was nearly 30-fold increase compared to 0.03% in WT. TAG was clearly the predominant neutral lipid across the three developmental stages in L3 and L5, peaking at the senescent stage (Figure 3B). The accumulation of the two galactolipids, monogalactosyldiacylglycerol (MGDG) and digalactosyldiaclglycerol (DGDG), in transgenic leaves rose significantly at the flowering stage, with L5 displaying the highest MGDG content as 1.38% (DW), which was increased nearly 2-fold compared to WT (Figure 3C), but subsequently dropped to a lower level in the senescent stage (Figure 3E). DGDG accumulation showed a similar trend of change with MGDG during the development. Phospholipids including phosphotidylcholine (PC), phosphatidylethanolamine (PE), and phosphatidylglycerol (PG) were also correspondingly varied in L3 and L5, particularly at the flowering and senescent stages. Compared to WT, contents of PC were significantly increased in the two transgenic lines at the flowering stage, and PE and PG contents in L5 were nearly doubled (Figure 3C). At the senescent stage, only PC remained the higher level than WT, particularly in L3 (0.36% of DW), whereas PG was barely detectable (Figure 3E). Despite the highest production of TFA, no significant variation was observed in polar membrane lipids between WT and transgenic plants at mature stage (Figure 3D).

**FIGURE 4:**
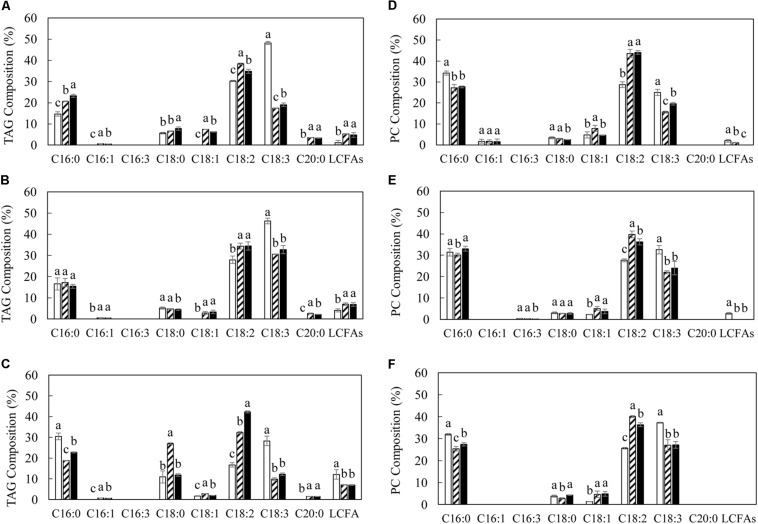
Fatty acid compositions of TAG and PC in potato leaves at the three developmental stages in WT (open bar), L3 (bar with upward diagonal) and L5 (black bar). **(A–C)** TAG at the flowering, mature, senescent stages respectively; **(D–F)** PC at the flowering, mature, senescent stages respectively. The data represent the mean values ± SD of three biological replicates. Letters (a, b, c, and d) above the bars are based on LSD, bars marked with different letters are statistically significantly different at *P* < 0.05.

**FIGURE 5:**
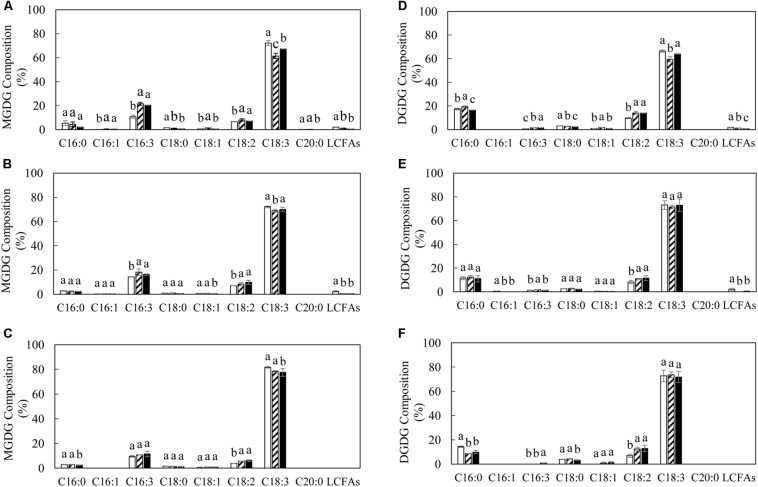
Fatty acid compositions of MGDG and DGDG in potato leaves at the three developmental stages in WT (open bar), L3 (bar with upward diagonal) and L5 (black bar). **(A–C)** MGDG at the flowering, mature, senescent stages respectively; **(D–F)** DGDG at the flowering, mature, senescent stages respectively. The data represent the mean values ± SD of three biological replicates. Letters (a, b, c, and d) above the bars are based on LSD, bars marked with different letters are statistically significantly different at *P* < 0.05.

The variation in TAG and polar membrane lipids in transgenic lines was accompanied by the alteration of fatty acid composition relative to WT (Figures 4, 5). The significant reduction in the level of α-linolenic acid (ALA, C18:3^Δ9,12,15^) in TAG and PC represented the major fatty acid change in transgenic potatoes compared to WT. Correspondingly, palmitoleic acid (C16:1^Δ9^), oleic acid (C18:1^Δ9^) and linoleic acid (LA, C18:2^Δ9,12^), as well as the long chain fatty acids (LCFAs) including arachidic acid (C20:0) and the others, were all increased, particularly in TAG at the flowering and mature stages (Figures 4A,B). However, a significant reduction in palmitic acid (C16:0) in TAG was observed in L3 and L5 at the senescent stage, and a 3-fold increase in stearic acid (C18:0) was particularly reflected in L3 (Figure 4C). By comparison, such distinct fluctuations in the levels of saturated fatty acids and monounsaturated fatty acids (MUFA) were not reflected in PC throughout the leaf development (Figures 4D–F). In galactolipids, the fatty acid composition of transgenic lines was mainly featured by the significantly increased hexadecatrienoic acid (C16:3) in MGDG at the flowering stage (Figure 5A), and enhanced LA in both galactolipids at all stages, relative to WT (Figure 5).

### Microscopic Observation of Potato Leaves Displayed Enlarged Cytosolic Lipid Droplets in Transgenic Lines and Plastoglobuli in Chloroplasts Throughout the Development

**FIGURE 6:**
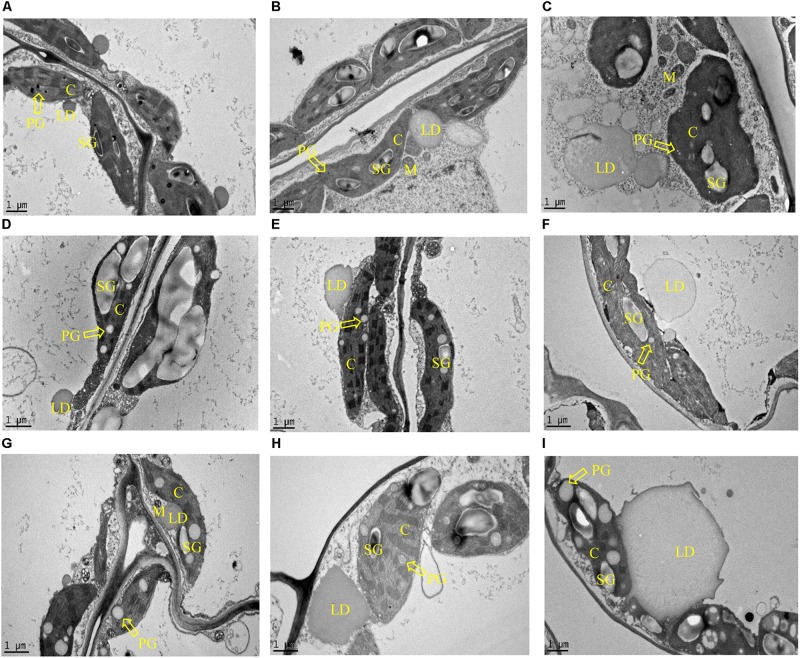
TEM imaging of the intracellular structures in potato leaves in the three developmental stages. Lipid droplet (LD), plastoglobuli (PG), chloroplast (C), starch granule (SG), and mitochondria (M) can be clearly visualized. The arrowheads point at PG. **(A–C)** Sections of leaf cells in WT, L3, and L5, respectively at the flowering stage; **(D–F)** Sections of leaf cells in WT, L3, and L5, respectively at the mature stage; **(G–I)** Sections of leaf cells in WT, L3, and L5, respectively at the senescent stage. PGs were observed in the chloroplast and LDs in the cytosol. L3 and L5 seem to display enlarged size but irregular morphology of LDs compared to WT, while PGs showed a similar trend of enlargement with LDs in plant development in both WT and the two transgenic lines. The scale bars correspond to 1 μm.

In parallel with the biochemical analysis, microscopic analysis of leaves sampled at the three developmental stages was undertaken. Both LD and plastoglobuli were observed in the mesophyll cells of potato leaves with the transmission electron microscopy (TEM) (Figure 6). LDs were found in the cytosol in all three samples: WT, L3 and L5, with proximity to chloroplast and mitochondria, and plastoglobuli were found inside the chloroplasts. Under the two-dimensional (2D) horizon, both LD and plastoglobuli spheres were visualized as irregular round shapes. The average diameters were therefore compared in order to reflect the possible variation in the morphology. In WT, LDs did not appear to vary significantly with the aging of the leaf, but plastoglobuli enlarged as leaves developed, the average diameter increasing more than ten times from less than 0.1 μm at the flowering stage to 1 μm in the senescent stage (Figures 6A,D,G). A similar observation in terms of plastoglobuli size increase was also made in L3 and L5. But, in addition to the expanding plastoglobuli, the transgenic mesophyll cells were featured with dramatically enlarged LDs often with irregular shapes. Particularly, at the flowering stage, the diameter of LDs in L3 and L5 were approximately 3 and 5 μm respectively, which was in sharp contrast to merely 1.5 μm in WT. At the senescent stage, LDs of the two transgenic lines had enlarged dramatically to about 10 μm in diameter (Figures 6H,I). However, the number of LDs did not show significant variation in L3 and L5 from WT when the scale bars were normalized to 1 μm in all the photographs. Starch granules were observed in abundance in all chloroplasts imaged. But compared to WT, L3 and L5 seemed to exhibit decreased numbers of starch granules and potential alteration in granule shapes (Figures 6E,F,H).

### Acyl Distribution in TAG and Galactolipids in the Senescent Potato Leaves and Lipid Compartmentalization

In order to explore the potential factors impacting TAG production in transgenic potato leaves, characterization of the positional distribution of fatty acids in lipids specifically distributed into chloroplast and cytosol was carried out, together with the fundamental analysis of the intracellular TFA allocation and identification of one of the major components, fatty acid phytyl ester, in plastoglobuli of plant chloroplasts at the senescent stage (Rottet et al., 2015). These experiments aimed at initially exploring the potential variation in lipid compartmentalization and plastoglobuli biogenesis in potato leaves.

TAG purified from the senescent leaves of WT and two transgenic lines were digested with the *Rhizopus arrhizus* lipase, which preferentially cleaves fatty acids bonded to the outer positions (*sn*-1 and *sn*-3) of the glycerol backbone of a TAG molecule, resulting in the production of hboxtextitsn-2 MAG and *sn*-1/3 FFA. Thus, the fatty acids in *sn*-2 MAG represent the fatty acids of *sn*-2 position of TAG and FFA molecules reflecting the acyl components of *sn*-1/3 positions. As displayed in Table 1, the relatively higher preference to outer positions by saturated fatty acids was observed in both WT and the transgenic lines, while unsaturated fatty acids, mainly the C18 polyunsaturated fatty acids (PUFA), showed a clear preference to the *sn*-2 position. The increase in LA largely at the expense of ALA in transgenic plants was also reflected on the *sn*-2 position. MGDG and DGDG were digested with the same lipase to yield lyso-MGDG and lyso-DGDG retaining the *sn*-2 acyl chain and releasing FFAs from the *sn*-1 position (Tables 2, 3). Similar to TAG, the unsaturated fatty acids, represented by C16:3, LA and ALA, showed preference to *sn*-2 position while saturated fatty acids, mostly palmitic acid and stearic acid, were enriched at *sn*-1 position.

**TABLE 1:** Positional distribution of fatty acids in TAG of potato senescent leaves.

**Sample**	**Position**	**C16:0**	**C16:1**	**C16:3**	**C18:0**	**C18:1^△^^9^**	**C18:1^△^^11^**	**C18:2**	**C18:3**	**C20:0**	**C20:2**	**C22:0**	**C24:0**
WT	Original	34.7	0.0	0.0	12.4	1.9	0.0	19.0	32.0	0.0	0.0	0.0	0.0
	sn-2	27.0	0.0	0.0	16.7	0.0	0.0	28.8	27.4	0.0	0.0	0.0	0.0
	sn-1/3	38.5	0.0	0.0	10.2	2.9	0.0	14.0	34.3	0.0	0.0	0.0	0.0
L3	Original	20.2	0.8	0.0	29.1	3.0	0.0	34.8	10.5	1.6	0.0	0.0	0.0
	sn-2	19.0	0.0	0.0	14.9	3.0	0.0	35.7	27.4	0.0	0.0	0.0	0.0
	sn-1/3	20.8	1.1	0.0	36.1	3.0	0.0	34.3	2.1	2.4	0.0	0.0	0.0
L5	Original	24.5	0.6	0.0	12.7	2.2	0.0	45.4	13.0	1.6	0.0	0.0	0.0
	sn-2	11.5	0.0	0.0	8.5	3.5	0.0	41.3	35.2	0.0	0.0	0.0	0.0
	sn-1/3	31.0	1.0	0.0	14.8	1.5	0.0	47.4	1.9	2.4	0.0	0.0	0.0

**TABLE 2:** Positional distribution of fatty acids in MGDG of potato senescent leaves.

**Sample**	**Position**	**C16:0**	**C16:1**	**C16:3**	**C18:0**	**C18:1^△^^9^**	**C18:1^△^^11^**	**C18:2**	**C18:3**	**C20:0**	**C20:2**	**C22:0**	**C22:2**	**C24:0**
WT	Original	2.8	0.0	9.5	1.5	0.7	0.0	3.8	81.7	0.0	0.0	0.0	0.0	0.0
	sn-1	16.5	0.0	0.0	19.1	0.0	0.0	5.0	59.4	0.0	0.0	0.0	0.0	0.0
	sn-2	6.8	0.0	19.1	8.6	0.0	0.0	5.7	59.8	0.0	0.0	0.0	0.0	0.0
L3	Original	2.8	0.0	10.6	1.4	0.9	0.0	5.6	78.7	0.0	0.0	0.0	0.0	0.0
	sn-1	18.5	0.0	0.0	23.8	1.2	0.0	8.2	48.3	0.0	0.0	0.0	0.0	0.0
	sn-2	9.8	0.0	24.0	13.2	0.0	0.0	10.2	42.8	0.0	0.0	0.0	0.0	0.0
L5	Original	2.4	0.0	11.6	1.5	0.8	0.0	6.1	77.6	0.0	0.0	0.0	0.0	0.0
	sn-1	13.8	0.0	0.0	14.2	1.2	0.0	9.2	59.8	1.0	0.0	0.8	0.0	0.0
	sn-2	7.2	0.0	26.0	10.3	0.9	0.0	10.1	45.5	0.0	0.0	0.0	0.0	0.0

**TABLE 3:** Positional distribution of fatty acids in DGDG of potato senescent leaves.

**Sample**	**Position**	**C16:0**	**C16:1**	**C16:3**	**C18:0**	**C18:1^△^^9^**	**C18:1^△^^11^**	**C18:2**	**C18:3**	**C20:0**	**C20:2**	**C22:0**	**C22:2**	**C24:0**
WT	Original	14.3	0.0	0.0	3.8	0.0	0.0	6.9	75	0.0	0.0	0.0	0.0	0.0
	sn-1	32.8	0.0	0.0	15.5	0.0	0.0	6.9	44.8	0.0	0.0	0.0	0.0	0.0
	sn-2	22.2	0.0	0.0	2.6	0.0	0.0	13.7	61.5	0.0	0.0	0.0	0.0	0.0
L3	Original	8.7	0.0	0.0	4.3	0.9	0.0	12.4	73.7	0.0	0.0	0.0	0.0	0.0
	sn-1	31.8	0.0	0.0	16.0	2.1	0.0	8.2	41.9	0.0	0.0	0.0	0.0	0.0
	sn-2	19.4	0.0	0.0	3.2	0.0	0.0	11.8	65.6	0.0	0.0	0.0	0.0	0.0
L5	Original	9.8	0.0	0.9	3.3	1.3	0.0	12.9	71.8	0.0	0.0	0.0	0.0	0.0
	sn-1	30.1	0.0	0.0	14.4	2.4	0.0	8.5	44.6	0.0	0.0	0.0	0.0	0.0
	sn-2	22.6	0.0	0.0	8.7	2.5	0.0	11.3	54.9	0.0	0.0	0.0	0.0	0.0

**FIGURE 7:**
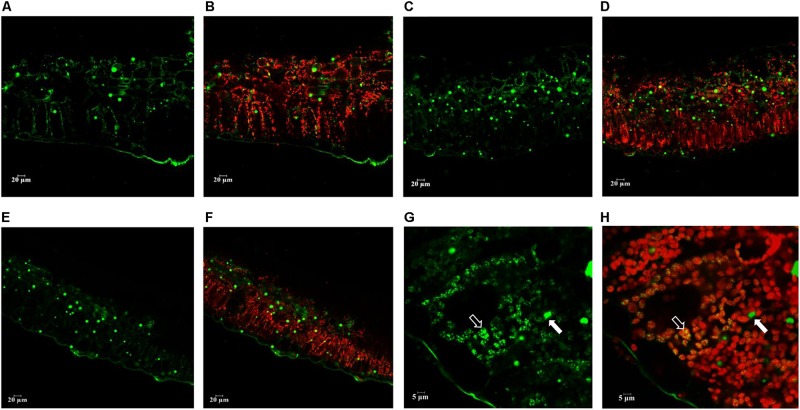
Confocal microscopy analysis of the neutral lipid droplets distribution in the senescent potato leaves. Neutral lipid droplets were stained with Bodipy (green), and the autofluorescence of chloroplasts was visualized in red. **(A,B)** Fresh leaf sections of WT; **(C,D)** Fresh leaf sections of L3; **(E,F)** Fresh leaf sections of L5; **(G,H)** Magnified leaf sections of L5 as an example. Plastoglobuli (marked in white open arrow head) and LD (marked in closed open arrow head) showed exclusive distribution in the cellular compartments, with plastoglobuli highly attached with the chloroplast and LD accumulated in the cytosol. The scale bars are located in the lower left corner for each photograph, images **(A–F)** (20 μm), images **(G,H)** (5 μm).

Confocal scanning microscopy was applied to visualize the distribution of neutral lipids in potato leaves at the senescent stage. The presence of neutral lipids mainly in the form of LD and plastoglobuli was visualized following Bodipy staining which is specific to neutral lipids (Figure 7). Compared with WT, significantly more abundant LDs were observed in L3 (Figures 7C,D) and L5 (Figures 7E,F). Plastoglobuli were associated with chloroplasts and appeared to be visually smaller than LD in cytosol. Such an observation was consistent with the TEM analysis. As being particularly exemplified in L5 with a further magnification, plastoglobuli, as a type of neutral lipids storage structure, were found to be overlapping with chloroplast, whereas LDs which incorporate TAG as the predominant component were found in cytosol (Figures 7G,H).

**FIGURE 8:**
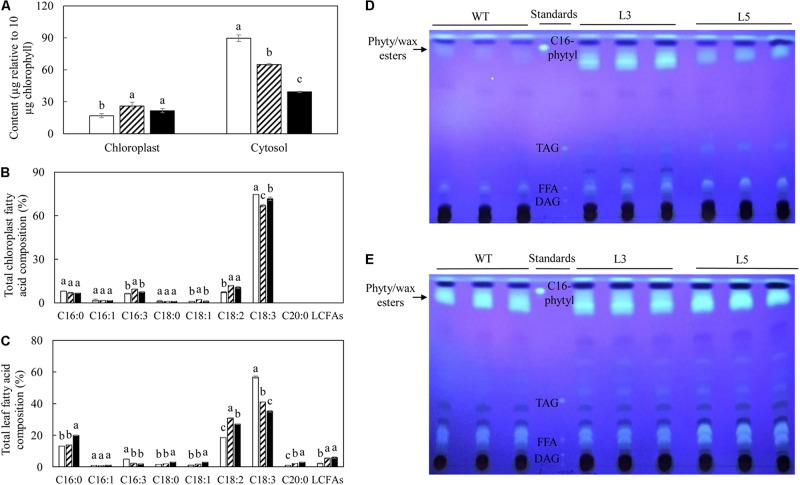
Acyl distribution analysis in the potato senescent leaves of WT (open bar), L3 (bar with upward diagonal) and L5 (black bar). **(A)** TFA contents normalized for the same amount of chlorophyll; **(B)** Total chloroplast fatty acid composition; **(C)** Total leaf fatty acid composition; **(D)** TLC fractionation of fatty acid phytyl esters at the flowering stage; **(E)** TLC fractionation of fatty acid phytyl esters at the senescent stage. TFA content in cytosol was calculated via subtracting the TFA of chloroplast from the TFA content in leaf; the palmitoyl hexadecanoate was synthesized as C16- phytyl standard to indicate the probable location of phytyl esters band on TLC, the wax ester was slightly mingled with the phytyl ester bands. The data represent the mean values ± SD of three biological replicates. Letters (a, b, c, and d) above the bars are based on LSD, bars marked with different letters are statistically significantly different at *P* < 0.05.

The acyl fatty acids derived from chloroplast and cytosol were then compared (Figure 8). After normalizing based on the amount of chlorophyll, the cytosolic TFA contents in potato leaves showed higher accumulation compared to the chloroplasts. Relative to WT, L3 and L5 showed a significant increase in the amount of TFA in chloroplasts but significant reduction in cytosols (Figure 8A). The fatty acid compositions of chloroplast and leaf were both featured by significantly increased LA at the expense of ALA in transgenic plants relative to WT, but the production of LCFAs was only identified in the leaf fatty acids (Figure 8C). Fatty acid phytyl esters, as one of the major components of plastoglobuli, were obtained by TLC fractionation, but slightly co-migrated with the wax ester components. At the flowering stage, significant deposition of the fatty acid phytyl esters was identified in both L3 and L5, in sharp contrast to WT in which the bands representing fatty acid phytyl esters were barely detectable on the TLC plate (Figure 8D), but became visible at the leaf senescent stage (Figure 8E).

### Effects of Transgene Expressions on Mature Potato Tubers

**FIGURE 9:**
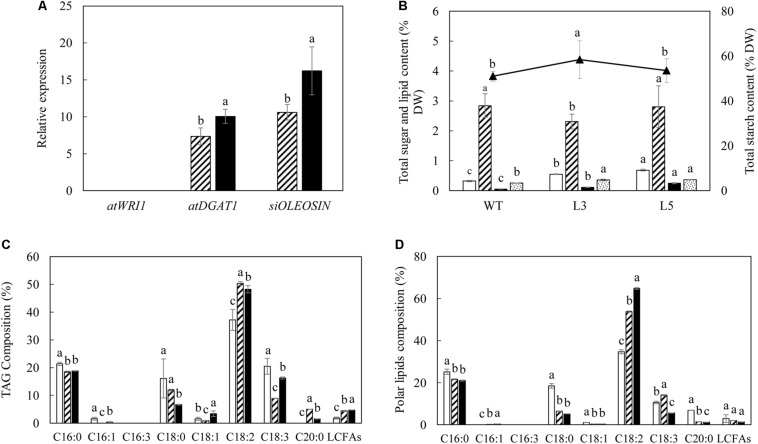
Lipid and carbohydrate analysis in the mature potato tubers. **(A)** Real-time qRT-PCR analysis of the transgene expressions in L3 (bar with upward diagonal) and L5 (black bar); **(B)** Contents of TFA (open bar), total soluble sugars (bar with upward diagonal), total starch (triangle marker), TAG (black bar) and total polar lipid (bar with small confetti); **(C)** Fatty acid composition of TAG in WT (open bar), L3 (bar with upward diagonal) and L5 (black bar); **(D)** Fatty acid composition of total polar lipids in WT (open bar), L3 (bar with upward diagonal) and L5 (black bar). The data represent the mean values ± SD of three biological replicates. Letters (a, b, c, and d) above the bars are based on LSD, bars marked with different letters are statistically significantly different at *P* < 0.05.

Mature potato tubers were sampled at the leaf senescent stage in parallel with leaves for analysis. Real-time PCR indicated that the transgenes of *AtDGAT1* and *SiOLEOSIN* also showed considerable levels of expression in the tubers of L3 and L5, whereas the expression of *AtWRI1* was barely identified (Figure 9A). Generally, the L5 tuber displayed the highest expression levels of the two transgenes relative to L3, with *SiOLEOSIN* being the most highly expressed. As a result, significant alteration in the contents of lipids and carbohydrates was observed. In particular, TFA content of L5 was doubled and TAG increased 5-fold compared to WT (Figure 9B). Similarly in L3, the TAG content increased by 2-fold. The total polar lipids which accounted for the major part of tuber TFA also increased significantly in both L3 and L5 relative to WT. Interestingly, in terms of the total carbohydrate variation, L3 showed significant increase in starch content and reduction in the total soluble sugars compared to WT, but not in L5 (Figure 9B). The fatty acid compositions of TAG (Figure 9C) and total polar lipids (Figure 9D) in the transgenic tubers were generally consistent with the transgenic leaves, except that L3 showed enhanced ALA in polar lipids relative to WT.

The preliminary assessment of several agronomic traits of potato tubers including density, yield, water content, the number of tubers per plant, and tuber size were tabulated in Supplementary Table 1, with photographs recorded of the mature and healthy tubers harvested from individual plants (Supplementary Figure 1). Significantly, tuber density and tuber water content of L3 and L5 were both reduced compared to WT, accompanied by relatively increased tuber yields (recorded as total fresh tuber weight). However, the number of tubers produced per plant, as well as the tuber size, has been significantly reduced in L3. Tubers were divided into four size groups (Size A, B, C, D) from the large size to small size by the maximum tuber length per potato. Tubers clustered in Size C (3 cm < tuber length < 6 cm) represented the largest proportion in WT and L5 (51.26 and 40.65%, respectively), while the Size A (tuber length > 9 cm) represented the largest proportion in L3 (34.38%). Accordingly, L3 showed the highest average weight of a single tuber, compared to WT and L5.

## Discussion

Plant leaves are the predominant source organ supporting high plant biomass establishment (Badeck et al., 2005; Ainsworth and Bush, 2011; Yaseen et al., 2013). Previous research on metabolic engineering of TAG in plant vegetative tissues were carried out in both model plants such as Arabidopsis (Fan et al., 2013, 2014) and tobacco (Vanhercke et al., 2014a, 2017), and high biomass crops such as sugarcane (Zale et al., 2016), potato tuber (Hofvander et al., 2016; Liu et al., 2017a), and sorghum (Vanhercke et al., 2018). This study applied the previously reported ‘Push, Pull and Protect’ genetic engineering strategy in potato leaf using the senescence-inducible promoter *SAG12* to drive the *AtWRI1* transcriptional factor, to minimize the undesirable effects of excessive expression of *WRI1* on plant development as most of the critical biological processes have been completed at the plant senescent stage (Gregersen et al., 2013; Avila-Ospina et al., 2014; Yang et al., 2015). The *CaMV-35S* promoter driving for constitutive gene expression was able to maintain a high level of *AtDGAT1* expression without causing substantial disturbance to plant development (Bouvier-Navé et al., 2000; Vanhercke et al., 2014a). *SSU* promoter is highly active in plant green tissues (Reiss et al., 1987) and used to control expression of the LD integral protein *SiOLEOSIN1* to assist in TAG packaging within the leaves.

Consistent with the observations made in transgenic tobacco leaf producing high levels of TAG (Vanhercke et al., 2014a, 2017), enhanced TAG accumulation in potato leaves was accompanied by total starch reduction and increase in soluble sugars, followed by varied equilibrium between neutral and polar lipids as well as altered fatty acid compositions. However, the increment of TAG varied significantly between these two *Solanaceae* species. The transgenic tobacco displayed 15% TAG of leaf DW (Vanhercke et al., 2014a), whereas in the transgenic potato leaves TAG was limited to just 0.8% of leaf DW (Figure 3A). In the high oil transgenic tobacco leaf, abundant LDs with large and irregular shapes were accumulated in the cytosol (Vanhercke et al., 2014a). However, in transgenic potato leaf, in addition to the significantly enlarged cytosolic LDs relative to WT under the 2D horizon, the increases in the number and size of plastoglobuli were also observed, which was in contrast to the observation made in transgenic tobacco leaf. Such discrepancies suggested that the three transgenes functioned in a similar pattern as in tobacco leaf, but clearly less effective in potato leaf. Although we could not rule out the possibility of insertion loci of transgene cassettes into a relatively inactive region of chromosome (Finnegan and McElroy, 1994; Kumar and Fladung, 2002; Bradford et al., 2005), it is more likely that the metabolic differences between tobacco and potato leaves is attributable to a certain extent. Potato leaves have evolved a system for rapid and efficient transfer of metabolites to stolon to support the underground tuber development (Fernie et al., 2002; Geigenberger, 2003; Roessner-Tunali et al., 2003). This is distinctive from tobacco leaf which has been selected as a highly productive organ (Vanhercke et al., 2014a).

Expression of the three transgenes has been discussed in transgenic potato tuber (Liu et al., 2017a) and tobacco leaf (Vanhercke et al., 2014a). In particular, *AtDGAT1* under the control of *CaMV-35S* promoter was the most highly expressed transgene, which has been viewed as a critical rate-limiting enzyme in TAG biosynthesis (Chapman et al., 2013; Xu and Shanklin, 2016). In this study, at the flowering stage, L3 had a significantly higher expression level of *AtDGAT1* than in L5 (Figure 1B), which was coincidental to a 2-fold higher TAG accumulation in L3 relative to L5 (Figure 2A). However, a significant boost of TAG synthesis in both transgenic lines was observed in the senescent stage, when the expression of *AtWRI1* was significantly increased. Despite the inconsistent expression levels of *SiOLEOSIN* (Figure 1B), similar levels of TAG accumulation were found at the senescent stage in both transgenic lines. The poor correlation between *SiOLEOSIN1* gene expression and TAG accumulation may suggest that the TAG production in transgenic potato leaves depends highly on the expression of *AtWRI1* and *AtDGAT1* transgenes, while *SiOLEOSIN* may not function as a major contributor. Further, the possibly enlarged size of LDs could have resulted from LD fusion and represented instability and vulnerability of LDs for degradation (Vanhercke et al., 2014a). The rapid rise in TAG accumulation in the senescent stage demonstrated the functionality of *AtWRI1* as a key gene in upregulating TAG biosynthesis with even lower expression levels relative to other transgenes (Maeo et al., 2009; Baud et al., 2009; To et al., 2012; Ma et al., 2015).

The alteration in leaf fatty acid compositions in L3 and L5 was consistent with the high oil transgenic tobacco leaf and potato tuber. The increase in LA and LCFAs and the reduction in ALA remained a hallmark of the ‘Push, Pull and Protect’ strategy, likely as the result of *AtWRI1* expression which impacts on the expression of fatty acid desaturases and fatty acid elongases (To et al., 2012; Liu et al., 2017a), from which other fatty acids such as the MUFA were affected. The expression of *SiOLEOSIN* may partially affect the membrane lipid constitution, for example PC, which is a major component of the phospholipids coating LDs (Chapman et al., 2012; Liu et al., 2017a). In transgenic potato lines, MGDG and DGDG were increased significantly at the flowering stage, but drastically reduced at the subsequent senescent stage. This could be due to the natural disintegration of the cellular membrane and chlorophyll catabolism which led to the degradation of photosynthetic membrane lipids, and further impact on the reallocation of plastidial acyl flux (Hölzl and Dörmann, 2019). The perturbation of membrane lipids not only affects to the compartmentalization of lipid metabolism, but also the biogenesis of plastoglobuli which are a class of unique lipoprotein organelle exclusively existing inside the chloroplast (Rey et al., 2000).

Plastoglobuli have been generally considered as a functional neutral lipids storage organelle which assists coordinating the lipid molecule exchanges between plastidial and extraplastidial envelopes of the chloroplast (Bréhélin and Kessler, 2008; Besagni and Kessler, 2013). A variety of neutral lipids can be synthesized in plastoglobuli, such as fatty acid phytyl esters, tocopherols, prenylquione, as well as TAG in some reports (Rottet et al., 2015; van Wijk and Kessler, 2017). The biogenesis of plastoglobuli was reported to be highly sensitive to various biotic/abiotic factors (Munné-Bosch et al., 2001; Munne-Bosch, 2005; Evans et al., 2010; Shao et al., 2016), and displayed a very similar structural morphology to the cytosolic LD (van Wijk and Kessler, 2017). The two transgenic potato lines, L3 and L5, have both displayed plastoglobuli in the chloroplast at the three developmental stages as observed by TEM, which was not observed in the high oil transgenic tobacco and sorghum leaves (Vanhercke et al., 2014a, 2017, 2018), despite some previous research on plastoglobuli on tobacco (Hurkman and Kennedy, 1975; Tevini and Steinmüller, 1985; Synková et al., 2006). Such a difference may therefore imply the potential particularity of potato leaf in terms of the lipid metabolism. Also, considering the similarity between plastoglobuli and LD, it has long been controversial whether chloroplasts harbor the capability to produce TAG directly or not (Fan et al., 2011; Moriyama et al., 2018). As in some microalga, for instance Chlamydomonas (*Chlamydomonas reinhardtii*), it was reported that TAG could accumulate in the chloroplast under some extreme conditions such as nitrogen starvation (Wang et al., 2009; Goodson et al., 2011; Simionato et al., 2013; Scranton et al., 2015).

However, according to the experimental results from the positional fatty acids distribution analysis (Tables 1–3), it was reflected that the positional fatty acid distribution pattern remained generally consistent among WT and the two transgenic plants, demonstrating that the TAG synthesis is dominated by the eukaryotic pathway, while the galactolipids were produced through the prokaryotic pathway (Kunst et al., 1989; Bates et al., 2009; Wang and Benning, 2012). As further reflected in the images of confocal microscopy (Figure 7H), it was obvious that the cellular distributions of LD and plastoglobuli were highly compartmentalized (Austin et al., 2006; Rottet et al., 2015; van Wijk and Kessler, 2017). Therefore, the incorporation of TAG within chloroplasts as neutral lipid droplets similar to plastoglobuli would be unlikely in potato leaves. Taken together, these results suggested that the biogenesis of plastoglobuli and LD in potato leaf is evidently differentiated. Nevertheless, possible competition of acyl flux may exist as suggested by the comparative fatty acid analysis between chloroplast and cytosol (Figure 8B), which may be mainly associated with the spatial regulation of different lipid gene expressions (Cernac and Benning, 2004; Shockey et al., 2006; Baud et al., 2009; Chapman and Ohlrogge, 2012). But the reduced fatty acids allocation in the cytosol of L3 and L5 could be the direct result of the carbon reallocation into plastoglobuli for fatty acid phytyl esters biosynthesis as displayed in the TLC fractionation (Figures 8D,E), possibly resulted from the enhanced expression of phytyl ester synthase (Lippold et al., 2012) as a result of the exogenous upregulation of *AtWRI1*.

Enhanced expressions of *AtDGAT1* and *SiOLEOSIN* as the result of transgene expression were also detected in mature transgenic potato tubers. However, the increase in TAG accumulation in L3 and L5 tubers did not show comparable values as reported in the high oil transgenic tubers regulated by a tuber-specific promoter (Liu et al., 2017a). We hypothesized that the lower level of TAG accumulation in transgenic tubers may be largely due to the invalid expression of *AtWRI1* with limited promoter function in tuber, but could be correlated with the *CaMV-35S* constitutive promoter controlled *AtDGAT1* expression (Figure 9A). By contrast, the *SiOLEOSIN* which was transcriptionally controlled by the *SSU* promoter and displayed the highest expression levels may not contribute to the TAG enhancement in tubers, which warrants for further investigation. Interestingly, L3 produced larger tubers with significantly increased starch content and reduced soluble sugars, in contrast to L5 in which the total carbohydrate content remained consistent with WT. Moreover, the reductions in tuber density and tuber water content compared to WT were observed in L3 and L5 (Supplementary Table 1), suggesting that the transgenes may have also potentially changed the tuber development to a certain extent, which could be largely due to the altered carbon partitioning in the source organ leaf (Sonnewald et al., 1997; Chincinska et al., 2008; Jonik et al., 2012; Katoh et al., 2015).

Plant lipid metabolism is highly regulated by a series of enzymatic steps and metabolic nodes, through the compartmentalization of different intracellular structures, the fatty acids produced from plastids are progressively and effectively distributed (Xu and Shanklin, 2016; Lavell and Benning, 2019). Genetic manipulation of the TAG metabolism thus requires great understanding on not only the carbon partitioning, but also how different lipid metabolic systems are orchestrated. Compared with other plant species, potato particularly reserves carbon in the underground tuber in which tuberization is a physiological process highly dependent on leaf (Fernie and Willmitzer, 2001; Timlin et al., 2006). In addition, concerning that the photosynthetic efficiency of chloroplast in senescent plant tissues may be weakened (Hensel et al., 1993; Paul and Pellny, 2003; Thomas, 2013), which could lead to the expansion of plastoglobuli, as well as the reduced capability of *de novo* fatty acids biosynthesis (Troncoso-Ponce et al., 2013; Rottet et al., 2015; van Wijk and Kessler, 2017; Hasan et al., 2019). Even though it has been known that TAG accumulation could be achieved in relatively higher level in the senescent leaf (Troncoso-Ponce et al., 2013), it was not typically reflected in the potato leaf in this study. Future studies could therefore be devoted to exploring the interrelationship between plastoglobuli and LDs in potato leaf cells as a model, as well as the carbon metabolic regulation between leaf and tuber in potato, in order to rationally design novel approaches for genetic enhancement of TAG accumulation in potato vegetative tissues.

### Experimental Procedures

#### Binary Plasmid Construct pOIL076

The binary plasmid construct pOIL076, which contains three transgene expression cassettes as *SAG12:AtWRI1*, *35S:AtDGAT1* and *SSU:SiOLEOSIN*, was designed by modification of our previously reported construct pJp3502 (Vanhercke et al., 2014a), by replacing the *SSU* promoter which controls *AtWRI1* with the *SAG12* promoter derived from Arabidopsis. Structure of the transgene cassettes in pOIL076 construct is summarized in Supplementary Figure 2.

#### Potato Transformation and Verification

Potato transformation was conducted following the method described in Liu et al. (2017a). The Phire plant direct PCR kit (Thermo Fisher Scientific, Waltham, MA, United States) was applied to quickly verify the presence of three transgenes in T_0_ potato leaves by following the manufacturer’s instructions. Primers used for the amplification of *AtWRI1* were: sense 5′-GCTTCCCATCTTCCGTTATG-3′, antisense 5′-GCAGAGG GTGACCAAAGAAG-3′; primers for *AtDGAT1* were: sense 5′-GGCGATTTTGGATTCTGCTGGC-3′, antisense 5′-GGAA CCAGAGAGAAGAAGTC-3′; and primers for *SiOLEOSIN* were: sense 5′-CAGCAGCAACAAACACGTG-3′, antisense 5′-GAGAAGATCACCAGGAGAG-3′. PCR reaction program included initial denaturation at 95°C for 3 min, followed with 40 cycles of 95°C for 10 s, 60°C for 30 s, 72°C for 30 s, which was carried out on a PCR machine (Thermo Fisher Scientific).

#### Selection of Representative Transgenic Lines for Characterization

A total of 17 transgenic plants were selected and grown alongside WT potato in a glasshouse (24/20°C, 16 h photoperiod). Potato leaves sampled at the senescent stage from the T_0_ transgenic population were screened for the TFA content, and two lines named L3 and L5 showing the most significantly increased TFA were selected for further analysis and synchronically propagated with WT under the same glasshouse environment for characterization. Samples for biochemical and molecular analysis were harvested at three potato developmental stages, starting from opening of its first flower as the flowering stage (>70% of flowers in one plant were blossoming), followed by mature (>80% of flowers in one plant were withered) and senescent (>50% aging leaves in one plant were visible) stages. Healthy and fully expanded leaves with the typical features of each developmental stage were collected from three randomly arranged and biologically replicated plants. Mature tubers were only sampled at the plant senescent stage. Samples were immediately freeze-dried for 72 h prior to extraction of lipids or RNA.

### Lipid Classes Characterization

The extraction of total lipids, lipid fractionation and quantification were carried out following the methods previously described by Liu et al. (2017a). Neutral lipids and free fatty acids (FFAs) were separated in the solvent system of hexane: diethyl ether: acetic acid (70: 30: 1, by volume) on TLC. Polar lipids including PC, MGDG, DGDG, PE, and PG were separated using the solvent system consisting of chloroform: methanol: acetic acid: distilled water (30: 5: 3: 1, by volume).

Positional distribution of fatty acids in TAG, MGDG and DGDG was investigated *via* lipase digestion. The *Rhizopus arrhizus* lipase (Fluka, Buchs, Switzerland) was utilized to digest TAG, MGDG and DGDG respectively. TAG, MGDG or DGDG were isolated from the total lipids of potato leaf at the senescent stage by TLC fractionation, and further purified using chloroform: methanol (2: 1, by volume). The treatment of each lipid digestion and subsequent fractionation were performed as described in Liu et al. (2017b). Fatty acid methyl esters (FAME) analysis was carried out using the samples prepared from the TLC purified *sn*-2 MAG from TAG, and the *sn*-1 FFA and *sn*-2 lyso-MGDG/DGDG from galactolipids by GC analysis. It should be noted that the fatty acid composition of the *sn*-1/3 FFA released from TAG was calculated as previously described in Christie et al. (1984). The digestion of galactolipids using the same methods as the TAG was previously reported by Yongmanitchai and Ward (1993).

Fatty acid phytyl esters were fractionated from the total leaf lipids on TLC using a solvent system hexane: diethyl ether: acetic acid (85: 15: 1, by volume) as described in Ischebeck et al. (2006). To indicate the probable position of fatty acid phytyl esters, the palmitoyl hexadecanoate was synthesized for use as the C16- phytyl standard in TLC analysis (Pereira et al., 2002).

### Total Starch and Soluble Sugars Measurement

Three phases were visibly separated in the chloroform/methanol/0.1M KCl based lipid extraction after centrifugation. The upper phase contains soluble sugars and proteins whereas the insoluble substances such as starch and fiber remain in the interphase and the lower phase containing the lipids dissolved in chloroform, which were used for analysis of total soluble sugars, starch and lipids, respectively. The total soluble sugars was analyzed according to the anthrone coloration method. Briefly, 5–10 μL of supernatant obtained from the lipid extraction was isolated and boiled for 10 min in 500 μL anthrone solution (0.2% anthrone in 70% H_2_SO_4_), and measured under 630 nm for absorbance. Total starch content was measured by boiling the sample for 30 min in 0.2 M NaOH and neutralized with 4 μL glacial acetic acid prior to be analyzed with the Megazyme Total Starch Kit (Megazyme International Ireland, Bray, Ireland) following the manufacturer’s instruction.

### RNA Extraction and qRT-PCR Analysis of Transgene Expression

Total RNAs from potato leaf tissues at different developmental stages were extracted using the RNeasy Mini Kit (Qiagen, Hilden, Germany), followed by quality check on a Nanodrop spectrophotometer ND 1000 (Thermo Fisher Scientific) and in an ethidium bromide-stained 1% agarose gel electrophoresis. The total RNA from mature potato tubers were extracted by the cetyl trimethyl ammonium bromide (CTAB) method (Reynolds et al., 2019) and purified using a RNeasy MinElute Cleanup Kit (Qiagen) following manufacturer’s instructions.

Expression of transgenes was analyzed using the qRT-PCR in triplicate biological samples, each with two technical replicates. Reverse transcription of total RNAs into cDNA was performed with a SuperScript^TM^ IV First-Strand Synthesis System kit (Thermo Fisher Scientific, Waltham, MA, United States). The oligo nucleotide sequence used for each transgene was as same as described in the PCR verification of the T_0_ transgenic potato plants. The reference gene and its corresponding primers selected to calibrate the expression analysis was the *S. tuberosum CYCLOPHILIN* (*stCYP*), as previously described in Liu et al. (2017a). The FastStart Universal SYBR Green Master (ROX) kit (Roche, Indianapolis, IN) was utilized to conduct the real-time qRT-PCR reaction, with the reaction program as 95°C for 3 min, 39 cycles of 95°C for 10 s, 58°C for 30 s and 72°C for 30 s using a Biorad 96 well PCR machine (Bio-Rad, Hercules, CA, United States). Calculation was made by following the 2^–Δ^ ^Δ^ ^Ct^ method (Livak and Schmittgen, 2001).

### Chloroplast Isolation, Purification and Fatty Acids Analysis

Chloroplasts were isolated from the senescent potato leaves following the method described in Hosaka and Hanneman (1987), the collected chloroplast pellets were gathered for the subsequent purification by using a sugar-gradient centrifuging method (Elias and Givan, 1978), through which intact potato chloroplasts were well separated and stored in 4 mL stock buffer (0.33 M sucrose in extraction buffer C) under 4°C. The integrity and quality of chloroplast was checked by light microscopy using Leica-DMR (Leica Microsystems, Wetzlar, Germany) (Supplementary Figure 3).

For the TFA extraction, 1.5 mL stock buffer containing purified chloroplasts on ice was first mixed with 0.5 mL 1M KCl solution, then 6 mL chloroform: methanol (2: 1, by volume) was added following 5 min vortex and 5 min centrifugation at 1,700 rpm. The lower phase was collected and evaporated under nitrogen, then dissolved in 200 μL chloroform as stock under −20°C. As chloroplast cannot be accurately quantified as leaf DW, the amount of chlorophyll was used as the reference standard for normalization. Chlorophyll from chloroplast and leaf lipid solutions was measured via the spectrometric method described in Warren (2008). FAME analysis of TFA were then proceeded using GC. The TFA in cytosol was calculated via subtracting the TFA of chloroplast from the TFA content in leaf.

### Microscopy Observation of Lipid Droplet

Transmission Electron Microscopy was applied to visualize the cellular distribution and morphology of LDs in potato leaf tissues. Dissected tissues were submerged in fixative 2.5% glutaraldehyde and 4% paraformaldehyde in 0.1 M phosphate buffer, and post fixed with 1% osmium tetroxide for 2 h. Fixed samples were infiltrated and embedded in LR white resin after gradual ethanol dehydration. 70 nm ultrathin sections were prepared with a Leica EM UC6 Ultramicrotome (Leica Microsystems). Each section was stained with 2% UA for 15 and 5 min with lead citrate. The sections were examined with a Hitachi H7100 transmission electron microscope (Hitachi, Tokyo, Japan) at 75 kV accelerating voltage. Confocal scanning microscopy analysis was processed by using freshly sampled potato leaf at the senescent stage, as described in Vanhercke et al. (2018).

### Basic Tuber Physiology Analysis

For the basic tuber physiological trait analysis, three healthy independent potato plants as biological replicates of WT, L3 and L5 respectively were measured. After harvesting all the mature tubers from an individual plant, the maximum tuber length was measured and divided into four groups according to the size (Size A, tuber length > 9 cm; Size B, 6 cm < tuber length < 9 cm; Size C, 3 cm < tuber length < 6 cm; Size D, tuber length < 3 cm). From each group, an intact tuber was selected then analyzed with the tuber water percentage (tuber water content/tuber fresh weight) and tuber density (fresh tuber weight/tuber volume). The tuber water content was calculated with the oven-drying method, and the tuber volume with the dewatering method.

### Statistical Analysis

GenStat 9.0 software was used to calculate the least significant difference (LSD) value of all data for multiple comparison.

## Data Availability Statement

The datasets generated for this study are available on request to the corresponding author.

## Author Contributions

XX designed the research, performed the experiments, and wrote the manuscript. SA and DH assisted the preparation of the transgenic construct and the tissue culture transformation. PS assisted the fatty acid positional distribution analysis. RD assisted the fatty acid phytyl ester analysis. LV assisted the confocal microscopic analysis. JL and MR assisted the TEM analysis. LT assisted the glasshouse maintenance. TV and SS provided precious guidance all along the research as project supervisors. PJS, ZL, and QL conceived and designed the project, and improved the manuscript.

## Conflict of Interest

The authors declare that the research was conducted in the absence of any commercial or financial relationships that could be construed as a potential conflict of interest.

## References

[B1] Ainsworth E. A. Bush D. R. (2011). Carbohydrate export from the leaf: a highly regulated process and target to enhance photosynthesis and productivity. *Plant Physiol.* 155 64–69. 10.1104/pp.110.167684 20971857 PMC3075787

[B2] Athanikar G. Badar P. (2016). Potato leaf diseases detection and classification system. *IJCSMC* 5 76–88.

[B3] Austin J. R. Frost E. Vidi P. A. Kessler F. Staehelin L. A. (2006). Plastoglobules are lipoprotein subcompartments of the chloroplast that are permanently coupled to thylakoid membranes and contain biosynthetic enzymes. *Plant Cell* 18 1693–1703. 10.1105/tpc.105.039859 16731586 PMC1488921

[B4] Avila-Ospina L. Moison M. Yoshimoto K. Masclaux-Daubresse C. (2014). Autophagy, plant senescence, and nutrient recycling. *J. Exp. Bot.* 65 3799–3811. 10.1093/jxb/eru039 24687977

[B5] Badeck F. W. Tcherkez G. Nogues S. Piel C. Ghashghaie J. (2005). Post-photosynthetic fractionation of stable carbon isotopes between plant organs - a widespread phenomenon. *Rapid Commun. Mass Spectrom.* 19 1381–1391. 10.1002/rcm.1912 15880634

[B6] Bates P. D. Durrett T. P. Ohlrogge J. B. Pollard M. (2009). Analysis of acyl fluxes through multiple pathways of triacylglycerol synthesis in developing soybean embryos. *Plant Physiol.* 150 55–72. 10.1104/pp.109.137737 19329563 PMC2675710

[B7] Baud S. Wuillème S. To A. Rochat C. Lepiniec L. (2009). Role of *WRINKLED1* in the transcriptional regulation of glycolytic and fatty acid biosynthetic genes in *Arabidopsis*. *Plant J.* 60 933–947. 10.1111/j.1365-313X.2009.04011.x 19719479

[B8] Besagni C. Kessler F. (2013). A mechanism implicating plastoglobules in thylakoid disassembly during senescence and nitrogen starvation. *Planta* 237 463–470. 10.1007/s00425-012-1813-9 23187680

[B9] Bourgis F. Kilaru A. Cao X. Ngando-Ebongue G. F. Drira N. Ohlrogge J. B. (2011). Comparative transcriptome and metabolite analysis of oil palm and date palm mesocarp that differ dramatically in carbon partitioning. *Proc. Natl. Acad. Sci. U.S.A.* 108 12527–12532. 10.1073/pnas.1106502108 21709233 PMC3145713

[B10] Bouvier-Navé P. Benveniste P. Oelkers P. Sturley S. L. Schaller H. (2000). Expression in yeast and tobacco of plant cDNAs encoding acyl CoA: diacylglycerol acyltransferase. *Eur. J. Biochem.* 267 85–96. 10.1046/j.1432-1327.2000.00961.x 10601854

[B11] Bradford K. J. Van Deynze A. Gutterson N. Parrott W. Strauss S. H. (2005). Regulating transgenic crops sensibly: lessons from plant breeding, biotechnology and genomics. *Nat. Biotechnol.* 23 439–444. 10.1038/nbt1084 15815671

[B12] Bréhélin C. Kessler F. (2008). The plastoglobule: a bag full of lipid biochemistry tricks. *J. Photochem. Photobiol.* 84 1388–1394. 10.1111/j.1751-1097.2008.00459.x 19067960

[B13] Brown A. P. Kroon J. T. Swarbreck D. Febrer M. Larson T. R. Graham I. A. (2012). Tissue-specific whole transcriptome sequencing in castor, directed at understanding triacylglycerol lipid biosynthetic pathways. *PLoS One* 7:e30100. 10.1371/journal.pone.0030100 22319559 PMC3272049

[B14] Carlsson A. S. Yilmaz J. L. Green A. G. Stymne S. Hofvander P. (2011). Replacing fossil oil with fresh oil - with what and for what? *Eur. J. Lipid Sci. Technol.* 113 812–831. 10.1002/ejlt.201100032 22102794 PMC3210827

[B15] Cernac A. Benning C. (2004). Wrinkled1 encodes an AP2/EREB domain protein involved in the control of storage compound biosynthesis in *Arabidopsis*. *Plant J.* 40 575–585. 10.1111/j.1365-313x.2004.02235.x 15500472

[B16] Chapman K. D. Dyer J. M. Mullen R. T. (2012). Biogenesis and functions of lipid droplets in plants thematic review series: lipid droplet synthesis and metabolism: from yeast to man. *J. Lipid Res.* 53 215–226. 10.1194/jlr.R021436 22045929 PMC3269164

[B17] Chapman K. D. Dyer J. M. Mullen R. T. (2013). Commentary, why don’t plant leaves get fat? *Plant Sci.* 207 128–134. 10.1016/j.plantsci.2013.03.003 23602107

[B18] Chapman K. D. Ohlrogge J. B. (2012). Compartmentation of triacylglycerol accumulation in plants. *J. Biol. Chem.* 287 2288–2294. 10.1074/jbc.R111.290072 22090025 PMC3268389

[B19] Chincinska I. A. Liesche J. Krügel U. Michalska J. Geigenberger P. Grimm B. (2008). Sucrose transporter *StSUT4* from potato affects flowering, tuberization, and shade avoidance response. *Plant Physiol.* 146 515–528. 10.1104/pp.107.112334 18083796 PMC2245842

[B20] Christie W. W. Clegg R. A. Calvert D. T. Noble R. C. (1984). The positional distributions of fatty acids in the triacylglycerols and phosphatidylcholines of the intestinal and popliteal lymph and plasma of sheep. *Lipids* 19 982–986. 10.1007/bf02534739 6527616

[B21] Costa G. G. Del Cardoso K. C. Bem L. E. Lima A. C. Cunha M. A. de Campos-Leite L. (2010). Transcriptome analysis of the oil-rich seed of the bioenergy crop *Jatropha curcas* L. *BMC Genome* 11:462. 10.1186/1471-2164-11-462 20691070 PMC3091658

[B22] Dita Rodriguez M. A. Brommonschenkel S. H. Matsuoka K. Mizubuti E. S. G. (2006). Components of resistance to early blight in four potato cultivars: effect of leaf position. *J. Phytopathol.* 154 230–235. 10.1111/j.1439-0434.2006.01089.x

[B23] Douches D. S. Kisha T. J. Coombs J. J. Li W. Pett W. L. Grafius E. J. (2001). Effectiveness of natural and engineered host plant resistance in potato to the Colorado potato beetle. *Hortscience* 36 967–970. 10.21273/hortsci.36.5.967 16686156

[B24] Durrett T. P. Benning C. Ohlrogge J. B. (2008). Plant triacylglycerols as feedstocks for the production of biofuels. *Plant J.* 54 593–607. 10.1111/j.1365-313X.2008.03442.x 18476866

[B25] Elias B. A. Givan C. V. (1978). Density gradient and differential centrifugation methods for chloroplast purification and enzyme localization in leaf tissue. *Planta* 142 317–320. 10.1007/BF00385083 24408195

[B26] Evans I. M. Rus A. M. Belanger E. M. Kimoto M. Brusslan J. A. (2010). Dismantling of *Arabidopsis thaliana* mesophyll cell chloroplasts during natural leaf senescence. *Plant Biol.* 12 1–12. 10.1111/j.1438-8677.2009.00206.x 20653883 PMC4383266

[B27] Fan J. Andre C. Xu C. (2011). A chloroplast pathway for the de novo biosynthesis of triacylglycerol in *Chlamydomonas reinhardtii*. *FEBS Lett.* 585 1985–1991. 10.1016/j.febslet.2011.05.018 21575636

[B28] Fan J. Yan C. Roston R. Shanklin J. Xu C. (2014). Arabidopsis lipins, *PDAT1* acyltransferase, and *SDP1* triacylglycerol lipase synergistically direct fatty acids toward β-oxidation, thereby maintaining membrane lipid homeostasis. *Plant Cell* 26 4119–4134. 10.1105/tpc.114.130377 25293755 PMC4247580

[B29] Fan J. Yan C. Zhang X. Xu C. (2013). Dual role for phospholipid: diacylglycerol acyltransferase: enhancing fatty acid synthesis and diverting fatty acids from membrane lipids to triacylglycerol in *Arabidopsis* leaves. *Plant Cell* 25 3506–3518. 10.1105/tpc.113.117358 24076979 PMC3809546

[B30] Fernie A. R. Tiessen A. Stitt M. Willmitzer L. Geigenberger P. (2002). Altered metabolic fluxes result from shifts in metabolite levels in sucrose phosphorylase - expressing potato tubers. *Plant Cell Environ.* 25 1219–1232. 10.1046/j.1365-3040.2002.00918.x

[B31] Fernie A. R. Willmitzer L. (2001). Molecular and biochemical triggers of potato tuber development. *Plant Physiol.* 127 1459–1465. 10.1104/pp.010764 11743089 PMC1540178

[B32] Finnegan J. McElroy D. (1994). Transgene inactivation: plants fight back! *Nat. Biotechnol.* 12 883–888. 10.1038/nbt0994-883

[B33] Fischer K. Weber A. (2002). Transport of carbon in non-green plastids. *Trends Plant Sci.* 7 345–351. 10.1016/s1360-1385(02)02291-4 12167329

[B34] Fleisher D. H. Timlin D. J. Reddy V. R. (2006). Temperature influence on potato leaf and branch distribution and on canopy photosynthetic rate. *Agron. J.* 98 1442–1452. 10.2134/agronj2005.0322

[B35] Geigenberger P. (2003). Regulation of sucrose to starch conversion in growing potato tubers. *J. Exp. Bot.* 54 457–465. 10.1093/jxb/erg074 12508056

[B36] Goodson C. Roth R. Wang Z. T. Goodenough U. (2011). Structural correlates of cytoplasmic and chloroplast lipid body synthesis in *Chlamydomonas reinhardtii* and stimulation of lipid body production with acetate boost. *Eukaryot. Cell* 10 1592–1606. 10.1128/EC.05242-11 22037181 PMC3232719

[B37] Graham I. A. (2008). Seed storage oil mobilization. *Annu. Rev. Plant Biol.* 59 115–142. 10.1146/annurev.arplant.59.032607.092938 18444898

[B38] Gregersen P. L. Culetic A. Boschian L. Krupinska K. (2013). Plant senescence and crop productivity. *Plant Mol. Biol.* 82 603–622. 10.1007/s11103-013-0013-8 23354836

[B39] Hasan M. M. Sharmeen I. A. Hakeem K. R. Alharby H. F. Hajar A. S. (2019). “The physiology and molecular biology of stress-induced senescence,” in *Senescence Signaling and Control in Plants*, 1st Edn, eds Sarwat M. Tuteja N. (Cambridge, MA: Academic Press), 1–14. 10.1016/b978-0-12-813187-9.00001-9

[B40] Hastilestari B. R. Lorenz J. Reid S. Hofmann J. Pscheidt D. Sonnewald U. (2018). Deciphering source and sink responses of potato plants (*Solanum tuberosum* L.) to elevated temperatures. *Plant Cell Environ.* 41 2600–2616. 10.1111/pce.13366 29869794

[B41] Hensel L. L. Grbić V. Baumgarten D. A. Bleecker A. B. (1993). Developmental and age-related processes that influence the longevity and senescence of photosynthetic tissues in *Arabidopsis*. *Plant Cell* 5 553–564. 10.1105/tpc.5.5.553 8518555 PMC160293

[B42] Higashi Y. Okazaki Y. Myouga F. Shinozaki K. Saito K. (2015). Landscape of the lipidome and transcriptome under heat stress in *Arabidopsis thaliana*. *Sci. Rep.* 5:10533. 10.1038/srep10533 26013835 PMC4444972

[B43] Hofvander P. Ischebeck T. Turesson H. Kushwaha S. K. Feussner I. Carlsson A. S. (2016). Potato tuber expression of *Arabidopsis WRINKLED1* increase triacylglycerol and membrane lipids while affecting central carbohydrate metabolism. *Plant Biotechnol. J.* 14 1883–1898. 10.1111/pbi.12550 26914183 PMC5069604

[B44] Hölzl G. Dörmann P. (2019). Chloroplast lipids and their biosynthesis. *Annu. Rev. Plant Biol.* 70 51–81. 10.1146/annurev-arplant-050718-100202 30786236

[B45] Hosaka K. Hanneman R. E. (1987). A rapid and simple method for determination of potato chloroplast DNA type. *Am. Potato J.* 64 345–353. 10.1007/bf02853596

[B46] Hurkman W. J. Kennedy G. S. (1975). Ultrastructural changes of chloroplasts in aging tobacco leaves. *Proc. Indiana Acad. Sci.* 85 89–95.

[B47] Ischebeck T. Zbierzak A. M. Kanwischer M. Dörmann P. (2006). A salvage pathway for phytol metabolism in *Arabidopsis*. *J. Biol. Chem.* 281 2470–2477. 10.1074/jbc.m509222200 16306049

[B48] Jonik C. Sonnewald U. Hajirezaei M. R. Flügge U. I. Ludewig F. (2012). Simultaneous boosting of source and sink capacities doubles tuber starch yield of potato plants. *Plant Biotechnol. J.* 10 1088–1098. 10.1111/j.1467-7652.2012.00736.x 22931170

[B49] Katoh A. Ashida H. Kasajima I. Shigeoka S. Yokota A. (2015). Potato yield enhancement through intensification of sink and source performances. *Breed. Sci.* 65 77–84. 10.1270/jsbbs.65.77 25931982 PMC4374566

[B50] Kelly A. A. Quettier A. L. Shaw E. Eastmond P. J. (2011). Seed storage oil mobilization is important but not essential for germination or seedling establishment in *Arabidopsis*. *Plant Physiol.* 157 866–875. 10.1104/pp.111.181784 21825108 PMC3192569

[B51] Klaus D. Ohlrogge J. B. Ekkehard Neuhaus H. Dormann P. (2004). Increased fatty acid production in potato by engineering of acetyl-CoA carboxylase. *Planta* 219 389–396. 15014998 10.1007/s00425-004-1236-3

[B52] Kong Q. Ma W. (2018). WRINKLED1 transcription factor: how much do we know about its regulatory mechanism? *Plant Sci.* 272 153–156. 10.1016/j.plantsci.2018.04.013 29807586

[B53] Kumar S. Fladung M. (2002). Transgene integration in aspen: structures of integration sites and mechanism of T-DNA integration. *Plant J.* 31 543–551. 10.1046/j.1365-313x.2002.01368.x 12182710

[B54] Kunst L. Browse J. Somerville C. (1989). Altered chloroplast structure and function in a mutant of *Arabidopsis* deficient in plastid glycerol-3-phosphate acyltransferase activity. *Plant Physiol.* 90 846–853. 10.1104/pp.90.3.846 16666887 PMC1061810

[B55] Lavell A. A. Benning C. (2019). Cellular organization and regulation of plant glycerolipid metabolism. *Plant Cell Physiol.* 60 1176–1183. 10.1093/pcp/pcz016 30690552 PMC6553661

[B56] Lippold F. vom Dorp K. Abraham M. Hölzl G. Wewer V. Yilmaz J. L. (2012). Fatty acid phytyl ester synthesis in chloroplasts of *Arabidopsis*. *Plant Cell* 24 2001–2014. 10.1105/tpc.112.095588 22623494 PMC3442583

[B57] Liu Q. Guo Q. Akbar S. Zhi Y. El Tahchy A. Mitchell M. (2017a). Genetic enhancement of oil content in potato tuber (*Solanum tuberosum* L.) through an integrated metabolic engineering strategy. *Plant Biotechnol. J.* 15 56–67. 10.1111/pbi.12590 27307093 PMC5253471

[B58] Liu Q. Wu M. Zhang B. Shrestha P. Petrie J. R. Green A. G. (2017b). Genetic enhancement of palmitic acid accumulation in cotton seed oil through RNAi down-regulation of ghKAS2 encoding β-ketoacyl-ACP synthase II (KASII). *Plant Biotechnol. J.* 15 132–143. 10.1111/pbi.12598 27381745 PMC5253470

[B59] Livak K. J. Schmittgen T. D. (2001). Analysis of relative gene expression data using real-time quantitative PCR and the 2 -ΔΔCT method. *Methods* 25 402–408. 10.1006/meth.2001.1262 11846609

[B60] Ma W. Kong Q. Grix M. Mantyla J. J. Yang Y. Benning C. (2015). Deletion of a C-terminal intrinsically disordered region of WRINKLED 1 affects its stability and enhances oil accumulation in *Arabidopsis*. *Plant J.* 83 864–874. 10.1111/tpj.12933 26305482

[B61] Maeo K. Tokuda T. Ayame A. Mitsui N. Kawai T. Tsukagoshi H. (2009). An AP2-type transcription factor, WRINKLED1, of *Arabidopsis thaliana* binds to the AW-box sequence conserved among proximal upstream regions of genes involved in fatty acid synthesis. *Plant J.* 60 476–487. 10.1111/j.1365-313X.2009.03967.x 19594710

[B62] Moriyama T. Toyoshima M. Saito M. Wada H. Sato N. (2018). Revisiting the algal “chloroplast lipid droplet”: the absence of an entity that is unlikely to exist. *Plant Physiol.* 176 1519–1530. 10.1104/pp.17.01512 29061905 PMC5813570

[B63] Munne-Bosch S. (2005). The role of α-tocopherol in plant stress tolerance. *J. Plant Physiol.* 162 743–748. 10.1016/j.jplph.2005.04.022 16008098

[B64] Munné-Bosch S. Jubany-Mari T. Alegre L. (2001). Drought-induced senescence is characterized by a loss of antioxidant defenses in chloroplasts. *Plant Cell Environ.* 24 1319–1327. 10.1046/j.1365-3040.2001.00794.x

[B65] Nguyen H. T. Silva J. E. Podicheti R. Macrander J. Yang W. Nazarenus T. J. (2013). Camelina seed transcriptome: a tool for meal and oil improvement and translational research. *Plant Biotechnol. J.* 11 759–769. 10.1111/pbi.12068 23551501

[B66] Noh Y. S. Amasino R. M. (1999). Identification of a promoter region responsible for the senescence-specific expression of SAG12. *Plant Mol. Biol.* 41 181–194. 10579486 10.1023/a:1006342412688

[B67] Paradiso R. Arena C. Rouphael Y. d’Aquino L. Makris K. Vitaglione P. (2018). Growth, photosynthetic activity and tuber quality of two potato cultivars in controlled environment as affected by light source. *Plant Biosyst.* 153 725–735. 10.1080/11263504.2018.1549603

[B68] Paul M. J. Pellny T. K. (2003). Carbon metabolite feedback regulation of leaf photosynthesis and development. *J. Exp. Bot.* 54 539–547. 10.1093/jxb/erg052 12508065

[B69] Peiman M. Xie C. (2006). Sensitive detection of potato viruses, PVX, PLRV and PVS, by RT-PCR in potato leaf and tuber. *Aust. Plant Dis. Notes* 1 41–46. 10.1016/j.jviromet.2009.06.027 19596379

[B70] Pereira A. S. Siqueira D. S. Elias V. O. Simoneit B. R. Cabral J. A. Neto F. R. A. (2002). Three series of high molecular weight alkanoates found in Amazonian plants. *Phytochemistry* 61 711–719. 10.1016/s0031-9422(02)00348-5 12423894

[B71] Poxleitner M. Rogers S. W. Lacey Samuels A. Browse J. Rogers J. C. (2006). A role for caleosin in degradation of oil-body storage lipid during seed germination. *Plant J.* 47 917–933. 10.1111/j.1365-313x.2006.02845.x 16961733

[B72] Reiss B. Wasmann C. C. Bohnert H. J. (1987). Regions in the transit peptide of SSU essential for transport into chloroplasts. *Mol. Genet. Genomics* 209 116–121. 10.1007/bf00329845 17186620

[B73] Rey P. Gillet B. Römer S. Eymery F. Massimino J. Peltier G. (2000). Over-expression of a pepper plastid lipid-associated protein in tobacco leads to changes in plastid ultrastructure and plant development upon stress. *Plant J.* 21 483–494. 10.1046/j.1365-313x.2000.00699.x 10758499

[B74] Reynolds K. B. Cullerne D. P. El Tahchy A. Rolland V. Blanchard C. L. Wood C. C. (2019). Identification of genes involved in lipid biosynthesis through *de novo* transcriptome assembly from *Cocos nucifera* developing endosperm. *Plant Cell Physiol.* 60 945–960. 10.1093/pcp/pcy247 30608545 PMC6498750

[B75] Roessner-Tunali U. Urbanczyk-Wochniak E. Czechowski T. Kolbe A. Willmitzer L. Fernie A. R. (2003). *De novo* amino acid biosynthesis in potato tubers is regulated by sucrose levels. *Plant Physiol.* 133 683–692. 10.1104/pp.103.024802 14512520 PMC219043

[B76] Rolando J. L. Ramírez D. A. Yactayo W. Monneveux P. Quiroz R. (2015). Leaf greenness as a drought tolerance related trait in potato (*Solanum tuberosum* L.). *Environ. Exp. Bot.* 110 27–35. 10.1016/j.envexpbot.2014.09.006

[B77] Rottet S. Besagni C. Kessler F. (2015). The role of plastoglobules in thylakoid lipid remodeling during plant development. *Biochim. Biophys. Acta* 1847 889–899. 10.1016/j.bbabio.2015.02.002 25667966

[B78] Scranton M. A. Ostrand J. T. Fields F. J. Mayfield S. P. (2015). Chlamydomonas as a model for biofuels and bio-products production. *Plant J.* 82 523–531. 10.1111/tpj.12780 25641390 PMC5531182

[B79] Shao R. Xin L. Zheng H. Li L. Ran W. Mao J. (2016). Changes in chloroplast ultrastructure in leaves of drought-stressed maize inbred lines. *Photosynthetica* 54 74–80. 10.1007/s11099-015-0158-6

[B80] Shockey J. M. Gidda S. K. Chapital D. C. Kuan J. C. Dhanoa P. K. Bland J. M. (2006). Tung tree *DGAT1* and *DGAT2* have nonredundant functions in triacylglycerol biosynthesis and are localized to different subdomains of the endoplasmic reticulum. *Plant Cell* 18 2294–2313. 10.1105/tpc.106.043695 16920778 PMC1560902

[B81] Simionato D. Block M. A. La Rocca N. Jouhet J. Maréchal E. Finazzi G. (2013). The response of *Nannochloropsis gaditana* to nitrogen starvation includes de novo biosynthesis of triacylglycerols, a decrease of chloroplast galactolipids, and reorganization of the photosynthetic apparatus. *Eukaryot. Cell* 12 665–676. 10.1128/EC.00363-12 23457191 PMC3647774

[B82] Sonnewald U. Hajirezaei M. R. Kossmann J. Heyer A. Trethewey R. N. Willmitzer L. (1997). Increased potato tuber size resulting from apoplastic expression of a yeast invertase. *Nat. Biotechnol.* 15 794–797. 10.1038/nbt0897-794 9255797

[B83] Synková H. Schnablová R. Polanská L. Hušák M. Šiffel P. Vácha F. (2006). Three-dimensional reconstruction of anomalous chloroplasts in transgenic ipt tobacco. *Planta* 223 659–671. 10.1007/s00425-005-0119-6 16160843

[B84] Tevini M. Steinmüller D. (1985). Composition and function of plastoglobuli. *Planta* 163 91–96. 10.1007/BF00395902 24249273

[B85] Thomas H. (2013). Senescence, ageing and death of the whole plant. *New Phytol.* 197 696–711. 10.1111/nph.12047 23176101

[B86] Timlin D. Lutfor Rahman S. M. Baker J. Reddy V. R. Fleisher D. Quebedeaux B. (2006). Whole plant photosynthesis, development, and carbon partitioning in potato as a function of temperature. *Agron. J.* 98 1195–1203. 10.2134/agronj2005.0260

[B87] To A. Joubès J. Barthole G. Lécureuil A. Scagnelli A. Jasinski S. (2012). WRINKLED transcription factors orchestrate tissue-specific regulation of fatty acid biosynthesis in *Arabidopsis*. *Plant Cell* 24 5007–5023. 10.1105/tpc.112.106120 23243127 PMC3556972

[B88] Troncoso-Ponce M. A. Cao X. Yang Z. Ohlrogge J. B. (2013). Lipid turnover during senescence. *Plant Sci.* 205-206 13–19. 10.1016/j.plantsci.2013.01.004 23498858

[B89] van Wijk K. J. Kessler F. (2017). Plastoglobuli: plastid microcompartments with integrated functions in metabolism, plastid developmental transitions, and environmental adaptation. *Annu. Rev. Plant Biol.* 68 253–289. 10.1146/annurev-arplant-043015-111737 28125283

[B90] Vanhercke T. Belide S. Taylor M. C. El Tahchy A. Okada S. Rolland V. (2018). Upregulation of lipid biosynthesis pathway increases the oil content in leaves of *Sorghum bicolor*. *Plant Biotechnol. J.* 17 220–232. 10.1111/pbi.12959 29873878 PMC6330533

[B91] Vanhercke T. Divi U. K. El Tahchy A. Liu Q. Mitchell M. Taylor M. C. (2017). Step changes in leaf oil accumulation via iterative metabolic engineering. *Metab. Eng.* 39 237–246. 10.1016/j.ymben.2016.12.007 27993560

[B92] Vanhercke T. Dyer J. M. Mullen R. T. Kilaru A. Rahman M. M. Petrie J. R. (2019). Metabolic engineering for enhanced oil in biomass. *Prog. Lipid Res.* 74 103–129. 10.1016/j.plipres.2019.02.002 30822461

[B93] Vanhercke T. El Tahchy A. Liu Q. Zhou X. Shrestha P. Divi U. K. (2014a). Metabolic engineering of biomass for high energy density, oilseed-like triacylglycerol yields from plant leaves. *Plant Biotechnol. J.* 12 231–239. 10.1111/pbi.12131 24151938 PMC4285938

[B94] Vanhercke T. El Tahchy A. Shrestha P. Zhou X. Singh S. P. Petrie J. R. (2013). Synergistic effect of *WRI1* and *DGAT1* coexpression on triacylglycerol biosynthesis in plants. *FEBS Lett.* 587 364–369. 10.1016/j.febslet.2012.12.018 23313251

[B95] Vanhercke T. Petrie J. R. Singh S. P. (2014b). Energy densification in vegetative biomass through metabolic engineering. *Biocatal. Agric. Biotechnol.* 3 75–80. 10.1016/j.bcab.2013.11.010

[B96] Wang Z. Benning C. (2012). Chloroplast lipid synthesis and lipid trafficking through ER-plastid membrane contact sites. *Biochem. Soc. Trans.* 40 457–463. 10.1042/BST20110752 22435830

[B97] Wang Z. Ullrich N. Joo S. Waffenschmidt S. Goodenough U. (2009). Algal lipid bodies: stress induction, purification, and biochemical characterization in wild-type and starchless *Chlamydomonas reinhardtii*. *Eukaryot. Cell* 8 1856–1868. 10.1128/EC.00272-09 19880756 PMC2794211

[B98] Warren C. R. (2008). Rapid measurement of chlorophylls with a microplate reader. *J. Plant Nutr.* 31 1321–1332. 10.1080/01904160802135092

[B99] Xu C. Shanklin J. (2016). Triacylglycerol metabolism, function, and accumulation in plant vegetative tissues. *Annu. Rev. Plant Biol.* 67 179–206. 10.1146/annurev-arplant-043015-111641 26845499

[B100] Xu X. Yang H. Singh S. P. Sharp P. J. Liu Q. (2018). Genetic manipulation of non-classic oilseed plants for enhancement of their potential as a biofactory for triacylglycerol production. *Engineering* 4 523–533. 10.1016/j.eng.2018.07.002

[B101] Yang M. F. Liu Y. J. Liu Y. Chen H. Chen F. Shen S. H. (2009). Proteomic analysis of oil mobilization in seed germination and postgermination development of *Jatropha curcas*. *J. Proteome Res.* 8 1441–1451. 10.1021/pr800799s 19152324

[B102] Yang Y. Munz J. Cass C. Zienkiewicz A. Kong Q. Ma W. (2015). Ectopic expression of *WRI1* affects fatty acid homeostasis in *Brachypodium distachyon* vegetative tissues. *Plant Physiol.* 169 1836–1847. 10.1104/pp.15.01236 26419778 PMC4634098

[B103] Yaseen M. Ahmad T. Sablok G. Standardi A. Hafiz I. A. (2013). Role of carbon sources for *in vitro* plant growth and development. *Mol. Biol. Rep.* 40 2837–2849. 10.1007/s11033-012-2299-z 23212616

[B104] Yongmanitchai W. Ward O. P. (1993). Positional distribution of fatty acids, and molecular species of polar lipids, in the diatom *Phaeodactylum tricornutum*. *Microbiology* 139 465–472. 10.1099/00221287-139-3-465 20050416

[B105] Zaheer K. Akhtar M. H. (2016). Potato production, usage, and nutrition - a review. *Crit. Rev. Food Sci. Nutr.* 56 711–721. 10.1080/10408398.2012.724479 24925679

[B106] Zale J. Jung J. H. Kim J. Y. Pathak B. Karan R. Liu H. (2016). Metabolic engineering of sugarcane to accumulate energy-dense triacylglycerols in vegetative biomass. *Plant Biotechnol. J.* 14 661–669. 10.1111/pbi.12411 26058948 PMC11388909

