# Supplementary material for: A Synergistic Genetic Engineering Strategy Induced Triacylglycerol Accumulation in Potato (Solanum tuberosum) Leaf
Source: Front Plant Sci. 2020 Mar 6;11:215. doi: 10.3389/fpls.2020.00215 (PMC7069356; doi:10.3389/fpls.2020.00215)
Supplement: Supplementary file 1 [file Data_Sheet_1.docx]

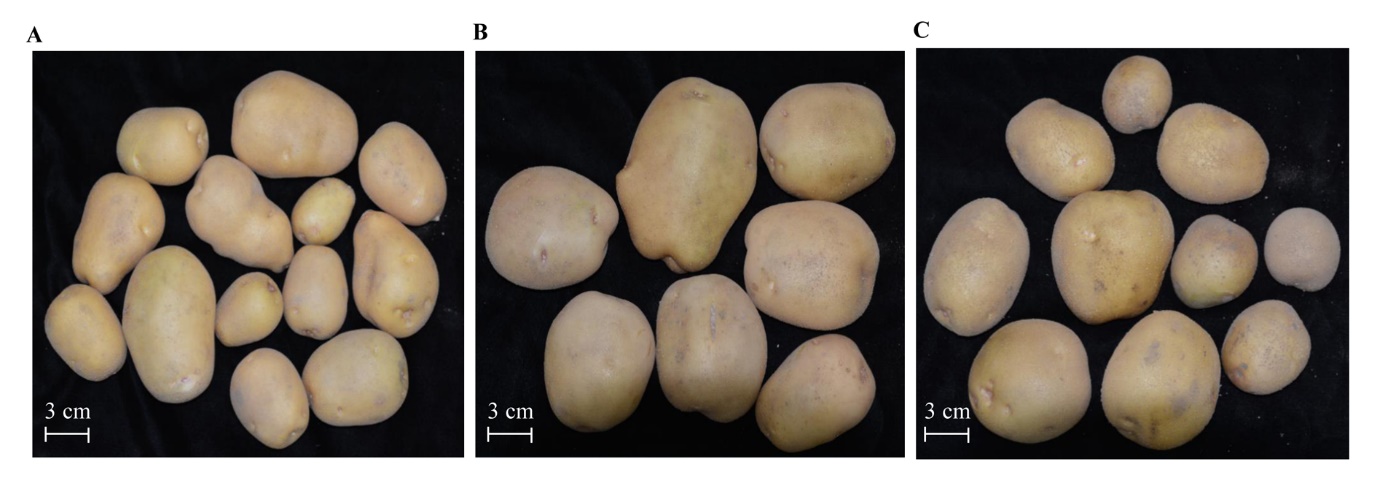


**Supplementary Figure 1.** Images of the freshly harvested mature potato tubers from a healthy individual plant at the plant senescent stage. (A) WT potato; (B) L3 potato; (C) L5 potato. Morphology of tubers were similar between WT and L5, but L3 displayed enlarged size and reduced tuber numbers. The scale bars correspond to 3 cm.


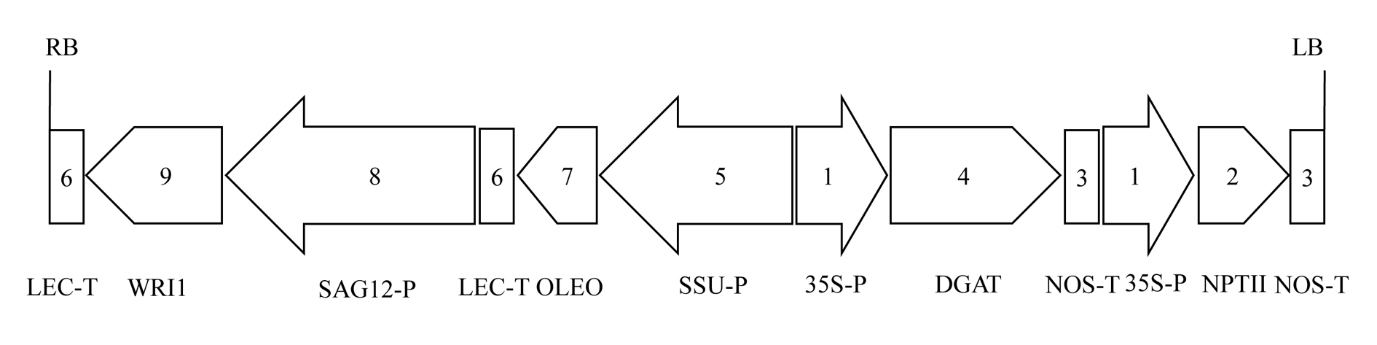


**Supplementary Figure 2.** Diagram of pOIL076 construct containing the binary T-DNA region with three transgene cassettes. Each configuration is represented as: 1, *CaMV-35S* promoter with duplicated enhancer region (35S-P); 2, NPTII; 3, nopaline synthase terminator (NOS-T); 4, *atDGAT1*; 5, *Arabidopsis thaliana* *SSU* promoter (SSU-P); 6, *Glycine max* lectin terminator (LEC-T); 7, *siOLEOSIN* (OLEO); 8, *A. thaliana* *SAG* promoter (SAG12-P); 9, *atWRI1* (WRI1)


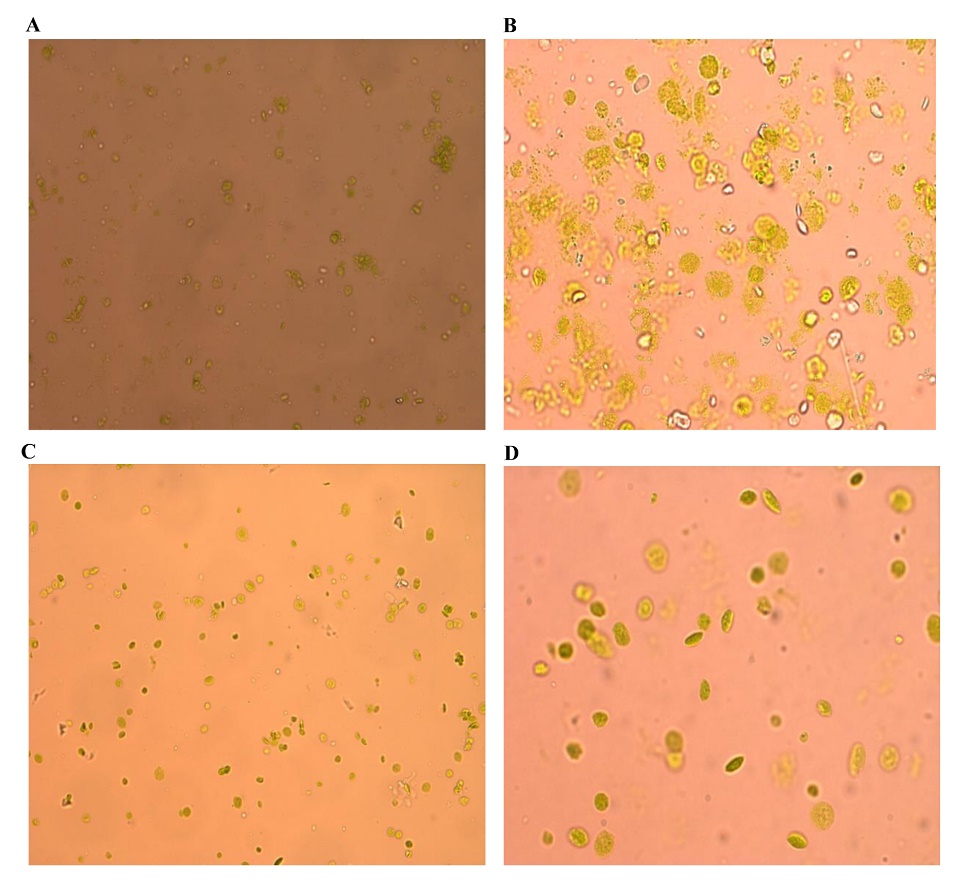


**Supplementary Figure 3.** Images of chloroplasts isolated and purified from the senescent leaf of L5 as an example. (A) Unpurified chloroplasts visualized under 20X lens of light microscopy; (B) Unpurified chloroplasts visualized under 100X lens of light microscopy; (C) Purified chloroplasts visualized under 20X lens of light microscopy; (D) Purified chloroplasts visualized under 100X lens of light microscopy. Debris and impurities can be clearly seen in the unpurified chloroplasts, while intactness and a neat background was reflected in the purified potato chloroplasts

**Supplementary Table 1.** Basic analysis of several important agronomic traits of potato tubers

| Line | Density (g/cm^3^) | Fresh weight (kg/per plant) | Water content (%) | Total number (per plant) | Number of size A  (per plant) | Number of size  B  (per plant) | Number of size  C  (per plant) | Number of size  D  (per plant) |
| --- | --- | --- | --- | --- | --- | --- | --- | --- |
| WT | 1.35 ± 0.07 ^a^ | 1.15 ± 0.03 ^c^ | 80.62 ± 1.26 ^a^ | 16.70 ± 0.33 ^a^  (100%) | 2.29 ± 0.22 ^a^  (13.71%) | 4.67 ± 1.25 ^a^  (27.96%) | 8.56 ± 1.68 ^a^  (51.26%) | 7.50 ± 1.14 ^a^  (44.91%) |
| L3 | 1.12 ± 0.02 ^b^ | 1.46 ± 0.04 ^b^ | 78.78 ± 1.31 ^b^ | 8.00 ± 0.49 ^b^  (100%) | 2.75 ± 0.68 ^a^  (34.38%) | 3.00 ± 0.61 ^b^  (37.5%) | 1.86 ± 0.16 ^b^  (23.25%) | 1.25 ± 0.24 ^b^  (15.63%) |
| L5 | 1.12 ± 0.03 ^b^ | 1.68 ± 0.02 ^a^ | 75.75 ± 4.31 ^b^ | 16.8 ± 0.30 ^a^  (100%) | 2.00 ± 0.39 ^a^  (11.90%) | 5.67 ± 1.07 ^a^  (33.75%) | 6.83 ± 1.32 ^a^  (40.65%) | 2.80 ± 0.88 ^b^  (16.67%) |

Data represented the mean value of sample ± SD, three replicate plants were analysed for WT, L3 and L5. Size A, tuber length > 9cm; Size B, 6cm < tuber length < 9 cm; Size C, 3 cm < tuber length < 6 cm; Size D, tuber length < 3 cm. Different letters are statistically significantly different at P < 0.05 between WT and two transgenic line
